# Advancing Arsenic Water Treatment Using UiO-66 and Its Functionalized Metal–Organic Framework Analogs

**DOI:** 10.3390/nano15211621

**Published:** 2025-10-24

**Authors:** Sangwoo Ji, Tarek M. Abdel-Fattah

**Affiliations:** 1 Geo-Environmental Research Division, Korea Institute of Geoscience and Mineral Resources (KIGAM), 124 Gwahak-ro, Yuseong-gu, Daejeon 34132, Republic of Korea; swji@kigam.re.kr; 2 Applied Research Center at Thomas Jefferson National Accelerator Facility and Department of Biology, Chemistry and Environmental Science, Christopher Newport University, Newport News, VA 23606, USA

**Keywords:** arsenic removal, UiO-66, metal–organic frameworks (MOFs), adsorption, water purification, defect engineering, magnetic composites

## Abstract

Arsenic contamination in water remains a critical global health challenge, affecting millions and causing severe diseases including cancer, skin lesions, and cardiovascular disorders. Adsorption using metal–organic frameworks (MOFs), particularly zirconium-based UiO-66 and its derivatives, offers a promising and sustainable approach for arsenic remediation due to their high surface area, tunable porosity, and strong chemical stability. Functionalized UiO-66 variants (e.g., –NH_2_, –SO_3_H, –COOH, –SH), metal-doped, or composite forms such as Fe_3_O_4_@UiO-66 exhibit arsenic adsorption capacities between 20 and 150 mg g^−1^, depending on synthesis and surface chemistry. Optimal adsorption occurs within pH 4–8, while high salinity or competing anions reduce performance by 15–40%. UiO-66 materials demonstrate excellent regeneration efficiency (70–95%) after multiple cycles, with limited metal leaching (1–3%). Advances through ligand functionalization, modulator-assisted synthesis, and composite integration have significantly improved adsorption capacity, selectivity, and reusability. However, challenges persist in achieving green, water-based synthesis, maintaining long-term stability under realistic water chemistries, and enabling scalable production. Future work should focus on eco-friendly fabrication, defect engineering, and mechanistic optimization to fully harness UiO-66’s potential as a high-performance and sustainable adsorbent for arsenic-contaminated water treatment.

## 1. Introduction

Arsenic contamination in water sources poses severe risks to both human health and the environment due to its high toxicity and persistence. It is estimated that over 150 million people worldwide are exposed to drinking water containing arsenic concentrations exceeding safe limits [1,2,3,4]. As a Group A human carcinogen, arsenic has been linked to a range of chronic health effects, and its widespread occurrence in groundwater and surface waters has resulted in a global public health crisis. The contamination of drinking and environmental waters has triggered large-scale outbreaks of arsenic poisoning, representing one of the most critical environmental health challenges faced worldwide [5,6].

Various methods have been developed for arsenic removal, including oxidation [7,8], coprecipitation [9], membrane filtration [10], ion exchange [11], coagulation [12], biological processes [13], and adsorption [13]. Among these methods, adsorption has emerged as a promising technology due to its low cost, simplicity, and potential applicability in individual household systems [14].

Metal–organic-frameworks (MOFs), innovative hybrid materials synthesized from inorganic metals and organic linkers, have captured considerable attention in recent years. These frameworks, characterized by the intricate coordination of metal clusters with organic ligands, constitute a distinct class of crystalline materials displaying a broad spectrum of structures, spanning from one-dimensional (1D) to three-dimensional (3D) architectures [15,16]. MOFs have emerged as exceptionally effective adsorbents in this context, thanks to their high surface area, customizable pore structures, and outstanding adsorption capacities. Extensive research has been dedicated to exploring numerous MOFs for arsenic removal, focusing on optimizing their performance and practical application in water treatment processes [17].

UiO-66, a zirconium-based metal–organic framework (MOF), emerged as a benchmark material owing to its exceptional water and chemical stability, high surface area, and tunable pore structure and chemistry through defect engineering and ligand functionalization. These attributes enable UiO-66 and its derivatives to excel across a broad range of applications, including adsorption, photocatalysis, gas and vapor capture, chemical sensing, and energy-related systems. Recent research continues to advance its versatility and performance. For instance, defect-rich UiO-66-NH_2_ demonstrated superior heavy-metal adsorption efficiency as a result of increased open coordination sites and enhanced surface functionality [18]. Similarly, the construction of S-scheme UiO-66-NH_2_/semiconductor heterostructures promoted efficient interfacial charge separation, improving photocatalytic hydrogen peroxide generation, nitrogen fixation, and overall photoactivity [19].

Defect-induced, hierarchically porous nanoscale MOFs have also been synthesized to optimize mass transfer and adsorption dynamics, markedly improving the uptake of volatile organic compounds (VOCs) [20]. Meanwhile, the anchoring of single-atom Cu species on UiO-66 frameworks has produced ultrasensitive surface-enhanced Raman scattering (SERS) sensors, enabling the rapid trace detection of volatile organic molecules [21]. Beyond catalysis and sensing, UiO-66-derived architectures have been employed to design advanced electrolyte and electrode interfaces for high-performance zinc-ion batteries, effectively facilitating ion transport and preventing dendrite growth [22].

Building on these demonstrated strengths, hydrolytic robustness, accessible Zr–OH coordination sites, and defect-tunable adsorption chemistry, UiO-66 and its functionalized analogs are exceptionally well-suited for arsenic adsorption and water purification. The framework’s stability under aqueous conditions, coupled with its ability to form inner-sphere complexes with arsenic oxyanions and respond to pH-dependent speciation, provides a strong foundation for developing next-generation adsorbents capable of selective, high-capacity arsenic removal under realistic environmental conditions [18,19,20,21]. Among the diverse range of MOFs, UiO-66 has attracted considerable attention for its potential as a highly efficient arsenic adsorbent. Ongoing research endeavors aim to elucidate its adsorption mechanisms, improve its efficiency, and simplify handling procedures [23].

In this review, we present a comprehensive and critical overview of recent advances in arsenic removal using UiO-66 and its functionalized derivatives, with particular emphasis on the strategies developed to enhance adsorption efficiency, stability, and operational practicality for water treatment applications.

## 2. Arsenic Contamination

Arsenic is a naturally occurring trace element present in the Earth’s crust at an average concentration of approximately 5 μg g^−1^ (ppm). Although it ranks around 54th in crustal abundance, arsenic can become locally enriched in certain regions due to natural mineralization processes. It is a constituent of more than 240 identified minerals, most commonly associated with sulfidic ores containing metals such as copper, gold, lead, and zinc [24]. Through natural geochemical and biological processes—including weathering, volcanic activity, and microbial mediation—arsenic can be mobilized and released into surrounding soils and water systems. Additionally, anthropogenic activities such as mining, ore smelting, fossil-fuel combustion, and groundwater extraction further contribute to its dispersion and elevate its concentration in the environment.

In natural aquatic systems, arsenic commonly occurs in two inorganic oxidation states, arsenite (As(III)) and arsenate (As(V)). Among these species, As(III) is generally more mobile, toxic, and difficult to remove than As(V), due to its neutral molecular structure and higher solubility. The predominant forms of inorganic arsenic in oxygen-rich waters are arsenate species, As(V) existing as H_3_AsO_4_, H_2_AsO_4_^−^, HAsO_4_^2−^, and AsO_4_^3−^—whereas arsenite species, As(III) occurring mainly as H_3_AsO_3_ and H_2_AsO_3_^−^, are more stable and abundant under anoxic or reducing conditions [2,25].

**Figure 1:**
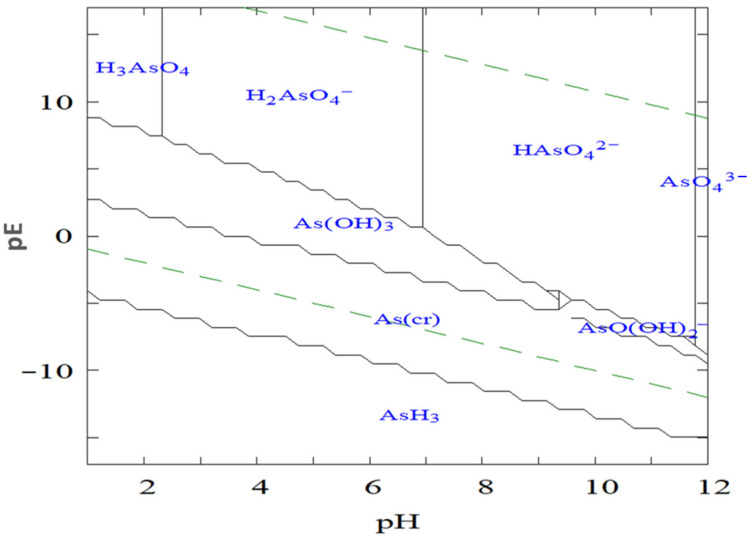
Logarithmic redox potential (pE)–pH diagram illustrating the stability domains of aqueous arsenic species in the AsO_4_^3−^–H_2_O system at 25 °C and 1 bar total pressure.

The log redox potential (pE) and pH of the surrounding environment are the key parameters controlling arsenic speciation, as illustrated in Figure 1. The pE value, which represents the electron activity of a solution, determines the prevailing oxidation state of arsenic under specific environmental conditions, higher pE values favor oxidized species such as arsenate (As(V)), whereas lower pE values promote reduced species such as arsenite (As(III)). In parallel, pH governs the degree of protonation and deprotonation of these species, thereby influencing their solubility, charge distribution, and overall chemical behavior. Figure 1 defines the stable pE–pH domains for the major aqueous arsenic species under varying redox and acidity conditions.

Under oxidizing conditions and at low pH (typically below 6.9), H_2_AsO_4_^−^ is the dominant species, while at higher pH values, HAsO_4_^2−^ becomes more prevalent. In strongly acidic or highly alkaline environments, H_3_AsO_4_ and AsO_4_^3−^ may also form, respectively. Conversely, under reducing conditions with pH values below approximately 9.2, the neutral H_3_AsO_3_ molecule predominates. This uncharged form is notably difficult to remove by conventional adsorption or coagulation processes due to its limited interaction with charged adsorbent surfaces.

The presence of arsenic in natural and drinking water sources represents a major global public health concern. Chronic exposure to elevated arsenic concentrations has been conclusively linked to a broad spectrum of carcinogenic and noncarcinogenic health effects, including cancers of the skin, bladder, and lungs, as well as cardiovascular diseases, hypertension, diabetes, and characteristic dermal lesions such as hyperkeratosis and pigmentation disorders [26].

**Figure 2:**
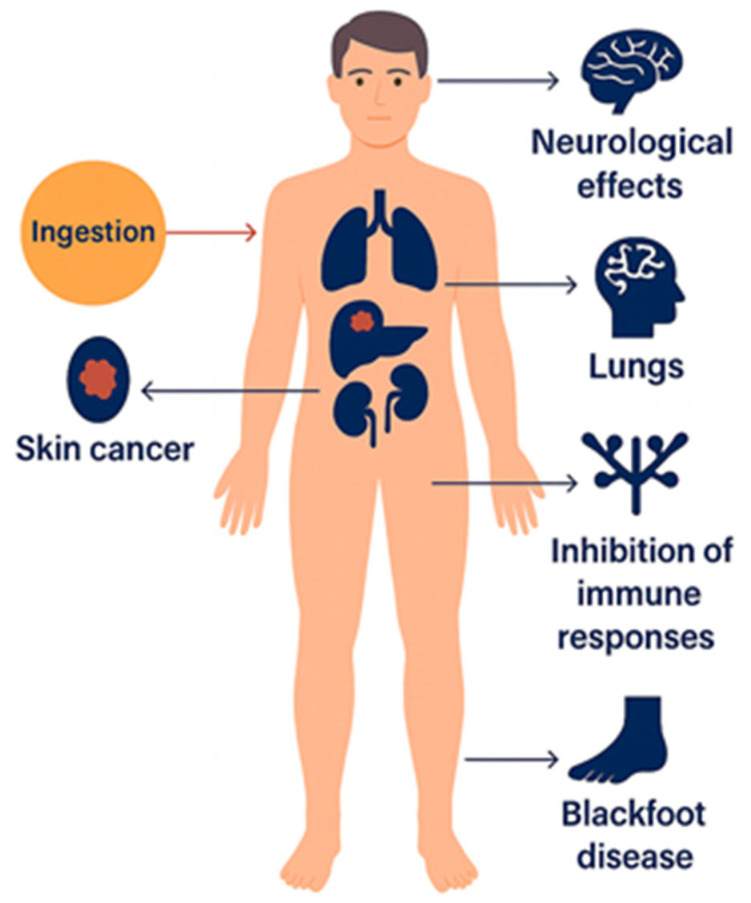
Health effects of inorganic arsenic exposure.

Epidemiological investigations conducted in regions with high arsenic exposure from drinking water, illustrated in Figure 2, have consistently demonstrated strong correlations between long-term ingestion and adverse health outcomes. The most severe and well-documented cases of arsenic poisoning and related cancers have been reported in Argentina, Bangladesh, Chile, China, Hungary, India (particularly West Bengal), Mexico, Romania, Taiwan, Vietnam, the United States, Nepal, Myanmar, and Cambodia, where groundwater contamination has led to widespread health impacts such as cancers, most severely bladder, lung, and skin cancers [6,27]. These findings collectively underscore the urgent need for effective monitoring and remediation strategies to mitigate arsenic exposure and its associated health risks.

**Figure 3:**
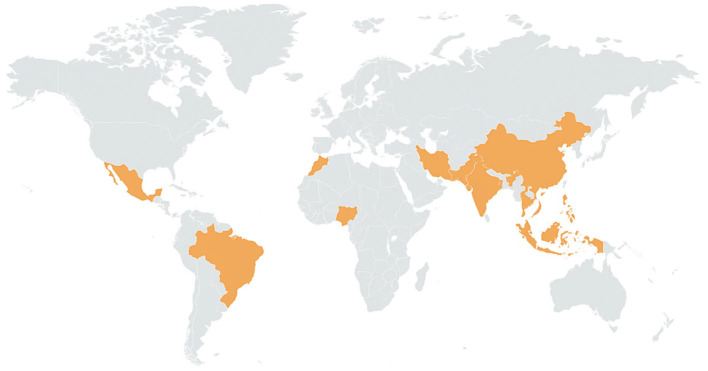
Global hotspots of arsenic contamination in groundwater. Yellow-shaded regions represent areas with documented high arsenic levels and associated health impacts from groundwater exposure.

In the Red River delta, Vietnam, groundwater is acutely contaminated by arsenic; up to 3000 μgL^−1^ of arsenic was detected in some places [28] as shown in Figure 3. Bengal Delta, one of the most serious areas of arsenicosis, has attracted intensive attention [29,30].

Although the adverse health impacts of arsenic exposure have been extensively documented, the underlying biochemical and molecular mechanisms responsible for its wide spectrum of toxic and carcinogenic effects remain complex and not yet fully elucidated [31].

Inorganic arsenic has been classified as a Group 1 human carcinogen by the International Agency for Research on Cancer (IARC) under the World Health Organization (WHO) and is widely recognized as one of the most toxic environmental contaminants. Following ingestion, arsenic distributes throughout the human body and can cause multiple adverse health effects, including skin cancer, organ damage (liver, kidney, and lungs), neurological impairment, immune suppression, and Blackfoot disease, a severe form of peripheral tissue necrosis [32,33].

To protect public health, the World Health Organization (WHO) established a guideline value of 10 μg L^−1^ for inorganic arsenic in drinking water in 1993, which was reaffirmed in the 2003 WHO Guidelines for Drinking-water Quality and remains in effect [34]. Similarly, the U.S. Environmental Protection Agency (US EPA) classifies inorganic arsenic as a known human carcinogen and, in January 2001, finalized a rule lowering the Maximum Contaminant Level (MCL) for arsenic in drinking water from 50 μg L^−1^ (set in 1942) to 10 μg L^−1^. The Maximum Contaminant Level Goal (MCLG), the level at which no known or anticipated health effects occur, was set at 0 μg L^−1^ due to arsenic’s carcinogenicity [35,36].

## 3. Arsenic Treatment Methods

**Figure 4:**
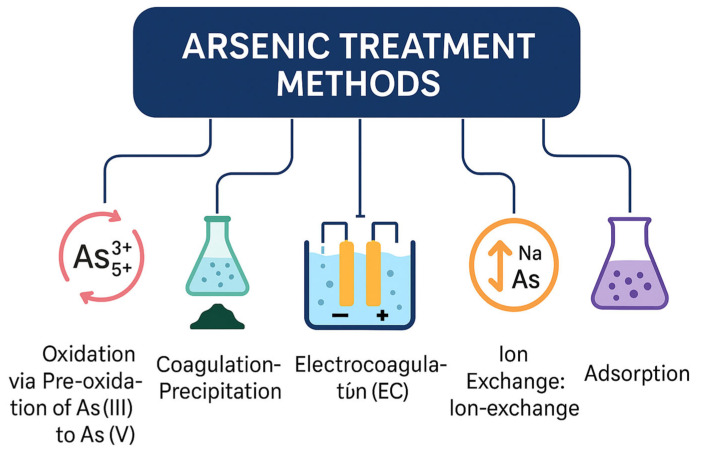
An overview of major arsenic treatment methods for water purification, illustrating six primary approaches: oxidation (pre-oxidation of As(III) to As(V)), coagulation–precipitation, electrocoagulation (EC), ion exchange, membrane-based separation, and adsorption. Each technique targets arsenic removal through distinct physicochemical mechanisms, contributing to effective mitigation of arsenic contamination in aqueous systems.

To combat arsenic contamination, various methods have been developed as shown in Figure 4.

Oxidation: The pre-oxidation of arsenite (As(III)) to arsenate (As(V)) is a crucial step to enhance the overall efficiency of arsenic removal processes [37]. The dominant arsenic species in groundwater depend on pH and redox potential, with As(III) typically prevailing under reducing conditions and As(V) under oxidizing environments [6]. Because As(III) is more mobile and less amenable to adsorption than As(V), its oxidation greatly improves subsequent immobilization or adsorption efficiency. The choice of oxidizing agents plays a key role in this transformation. Although natural aeration or oxygenation can promote partial oxidation, these methods are often slow and inefficient for rapid conversion. Therefore, chemical oxidants such as chlorine dioxide and chloramine [8], potassium permanganate (KMnO_4_ or α-MnO_2_) [38], and other reagents including ozone (O_3_), chlorine (Cl_2_), hydrogen peroxide (H_2_O_2_), and Fenton’s reagent are widely employed to ensure efficient pre-oxidation of As(III) to As(V) [39].

Coagulation–Precipitation: Coagulation is commonly used in drinking water treatment to destabilize dissolved and suspended solids allowing their aggregation to form flocs, which are subsequently removed via sedimentation [40]. Coagulation involves adding iron or aluminum salts to water to form flocs, which adsorb arsenic particles, facilitating their removal through filtration or sedimentation. Effective for eliminating both As(III) and As(V). Relatively simple process with low operational costs. pH and chemical dosage must be carefully controlled for optimal performance. Sludge disposal may pose environmental challenges.

Alum and Iron Precipitation: Traditional arsenic removal technologies often rely on chemical precipitation and coagulation using aluminum, iron, or zirconium salts, which effectively convert dissolved arsenic species into insoluble hydroxides that can be separated from water [12,41]. More recently, an advanced electrochemical approach known as air-cathode-assisted iron electrocoagulation (ACAIE) has been developed, enabling the in situ generation of hydrogen peroxide (H_2_O_2_) at the cathode to accelerate oxidation and coagulation reactions. This process significantly enhances arsenic removal efficiency while reducing chemical reagent consumption and sludge production [42].

Electrocoagulation (EC): The electrocoagulation process represents a promising electrochemical technique for the removal of arsenic from aqueous systems. In this method, sacrificial metal electrodes—commonly iron, aluminum, or zinc—serve as sources of coagulant species that are generated in situ through electrolytic oxidation of the anode upon the application of a direct current [43]. The efficiency of the EC process largely depends on the electrode material and solution pH, with iron and aluminum showing particularly high performance under optimized conditions. Compared with traditional chemical coagulation, EC typically achieves greater removal efficiency because the electrochemically generated metal hydroxides are more reactive and better dispersed. Moreover, partial oxidation of As(III) to As(V) may occur during the process, enhancing adsorption onto the freshly formed metal hydroxide flocs [44]. However, a key environmental consideration following EC treatment is the potential leaching of residual arsenic from the produced sludge, which necessitates proper handling and disposal.

Ion Exchange: Ion-exchange involves replacing arsenic ions in water with other ions present on an exchange medium, such as resin. Highly effective at removing arsenic, particularly at low concentrations. Can be used for both As(III) and As(V) removal. But, requires regeneration of the exchange medium, which can be costly and produce chemical waste. Performance may be affected by the presence of competing ions.

Membrane Techniques: Membrane-based separation is a widely applied technique for the removal of arsenic and other waterborne contaminants, utilizing synthetic materials containing billions of nanoscale pores that act as selective barriers, allowing water molecules to pass while restricting the transport of larger or undesired solutes [45]. The process operates under a pressure gradient between the feed and permeate sides, which drives water through the membrane matrix. Membrane filtration systems are broadly categorized into low-pressure processes, such as microfiltration (MF) and ultrafiltration (UF), and high-pressure processes, including nanofiltration (NF) and reverse osmosis (RO). Among these, reverse osmosis is particularly effective for arsenic removal, as the semi-permeable membrane enables selective rejection of both As(III) and As(V) species, achieving high purification efficiency even in heavily contaminated waters. However, the practical implementation of RO systems faces challenges, including high energy consumption, membrane fouling, and the requirement for frequent maintenance, all of which increase operational costs and system downtime. Moreover, the generation of concentrated wastewater (brine) during the RO process poses additional environmental concerns, underscoring the need for sustainable management and energy-efficient membrane designs.

Adsorption: A wide range of adsorbent materials have been employed for arsenic removal from water, including activated carbon, activated alumina, zero-valent iron, iron oxides, and natural clays. Among these, activated alumina (AA) and granular ferric oxides/hydroxides are regarded as the most effective adsorbents due to their high affinity for arsenic species, abundant availability, and operational simplicity [13]. At the household level, sand filtration systems have been successfully applied for more than three decades to remove As(III), as well as other co-occurring metals such as Fe(II) and Mn(II) from groundwater [46]. Within these filters, As(III), Fe(II), and Mn(II) undergo co-oxidation, leading to the formation and precipitation of As(V)-bearing Fe(III) (oxyhydr)oxides and Mn(III/IV) oxides on sand surfaces [47].

Iron-oxide-based materials have been demonstrated to be particularly effective in arsenic adsorption. For instance, Chowdhury and Yanful (2011) reported that magnetite (Fe_3_O_4_) nanoparticles exhibited a maximum adsorption capacity of 3.70 mg/g for both As(III) and As(V) [48]. In general, adsorption remains one of the most promising approaches for arsenic remediation owing to its simplicity, cost-effectiveness, and minimal secondary pollution. Numerous adsorbents have been developed for the simultaneous removal of multiple arsenic species, achieving varying adsorption capacities under different physicochemical conditions [49,50,51]. Representative examples are summarized in Table 1.

Among emerging materials, metal–organic frameworks (MOFs), also known as porous coordination polymers, have gained significant attention for arsenic decontamination. Their highly ordered and tunable pore structures, derived from metal-cluster nodes interconnected by organic linkers, allow precise control over surface chemistry and adsorption sites. Consequently, MOFs, and particularly UiO-66 and its functionalized derivatives, exhibit superior adsorption performance compared to traditional adsorbents and are now considered next-generation materials for efficient and selective arsenic removal [52].

**Table 1:** Some list of different adsorbents used for arsenic adsorption from water.

Adsorbents	Pollutants	Surface Area (BET) ** (m^2^ g^−1^)	$q_{max}$ *** (mg g^−1^)	References
Zero-valent Iron (ZVI)	As(V)	2.53	0.73	Su and Plus (2001) [53]
ZVI	As(V)	187	66.8	Peng et al. (2017) [49]
ZVI	As(V)	997	1.42	Zhang et al. (2021) [54]
Activated Carbon (AC)	As(V)	-	30.5	Mohan and Pittman (2007) [13]
AC (Coconut shell with 3% ash)	As(V)	1150–1250	2.4	Lorenzen et al. (1995) [55]
AC (Fe_3_O_4_-loaded)	As(V)	349	204.2	Liu et al. (2010) [56]
Organic biochar	As(V)	-	16.2	Zhu et al. (2016) [50]
TiO_2_	As(III)/As(V)	-	32.4/41.4	Bang et al. (2005) [57]
Laterite	As(III)/As(V)	181	8.0/24.8	Maiti et al. (2010) [58]
Hydrous ferric oxide/zeolite	As(V)	-	44.4	Habuda-Stanic et al. (2008) [59]
Fe-Mn-Al oxides/oxyhydroxides	As(III)/As(V)	456	34.3/21.2	Jian and Maiti (2022) [60]
Activated alumina (Al_2_O_3_-CeO_2_)	As(III)/As(V)	-	10.5/13.6	Nakamoto and Kobayashi (2019) [61]
Magnetite	As(III)/As(V)	60	29.1/11.4	Yean et al. (2005) [62]
Magnetite	As(III)/As(V)	-	18.7/17.2	Liu et al., (2015) [63]
Zr-VBZ *	As(III)/As(V)	421	6.5/7.0	Gupta et al. (2022) [64]
Amorphous ZrO	As(III)/As(V)	327	83/32.4	Cui et al. (2012) [65]
ZrO_2_-sawdust	As(III)/As(V)	-	29.0/12.0	Setyono and Valiyaveettil (2014) [66]
Fe-Mn binary oxide coating sands	As(III)/As(V)	117.4	22.07/11.94	Han et al. (2022) [67]
Fe_3_O_4_ coated wheat straw	As(III)/As(V)	-	3.9/8.1	Hao et al. (2015) [68]
Fe_3_O_4_ nanoparticles	As(III)/As(V)	179	16.56/46.06	Feng et al. (2012) [69]
Zr doped β-FeOOH nanoparticles	As(V)	330	120	Sun et al. (2013) [51]

* Zr-VBZ: zirconyl dimethacrylate-co-vinylbenzyl chloride, ** BET = Brunauer–Emmett–Teller surface area and *** Based on Langmuir Model.

## 4. The MOFs for Water Treatment

Metal–organic frameworks (MOFs) are highly porous materials with exceptional surface areas, allowing guest molecule encapsulation and broad applications [70,71]. Their tunable properties arise from precise control of metal clusters and organic linkers [72], supporting uses in catalysis, gas storage, sensing, and water treatment [73]. The concept evolved from early coordination networks [74] to modern MOFs [75,76,77,78]. Subsequent designs—MOF-5, ZIF-8, MIL-101, UiO-66—demonstrated high porosity [79,80,81,82] and improved water stability via strong M–L bonds [83,84].

**Figure 5:**
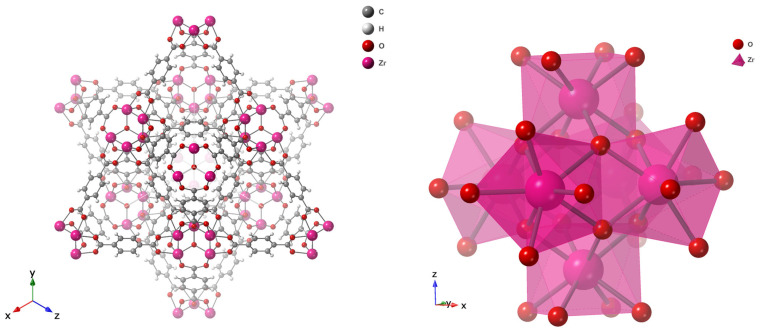
Unit Cell of UiO-66 (**right**) and the Octahedral Zr_6_O_8_ Core (**left**), which forms the primary building block of the structure.

**Figure 6:**
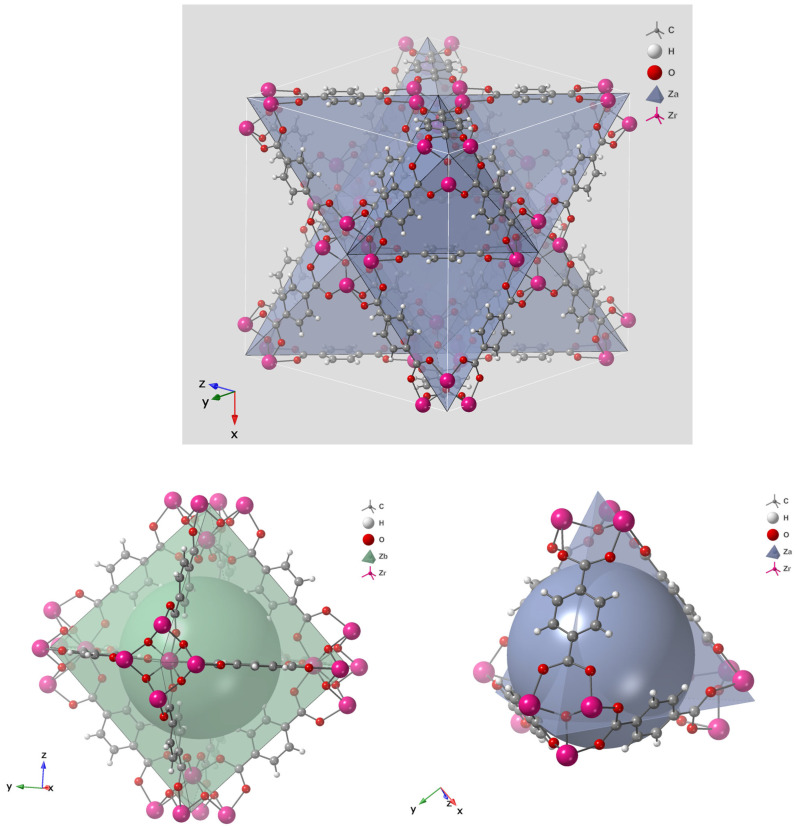
Polyhedral Structure of UiO-66 (**top**), Tetrahedral (Blue Sphere) and Octahedral (Green Sphere) Pore Geometries.

Early generations of MOFs exhibited poor water stability because their coordination bonds were susceptible to hydrolysis by strongly coordinating molecules such as water, leading to structural collapse [83,84]. Water-stable frameworks were later achieved by employing high-valence metal ions such as Zr^4+^, Al^3+^, and Cr^3+^, which form strong metal–carboxylate bonds [84]. Among these, UiO-66, developed by Lillerud’s group at the University of Oslo in 2008 [82], stands out for its exceptional chemical and thermal robustness. Its architecture is based on Zr_6_O_4_(OH)_4_ nodes connected by terephthalate (BDC^2−^) linkers, forming a face-centered cubic (fcc) topology as classified by the Reticular Chemistry Structure Resource (RCSR) [85]. The resulting framework contains tetrahedral cages (~8 Å), showed in Figure 6 as blue sphere and octahedral cages (~11 Å), as green sphere, interconnected through triangular windows (~6 Å), which facilitate efficient molecular diffusion and adsorption as in Figure 5 and Figure 6 [86]. The strong Zr–O–C coordination bonds and high connectivity (12-coordinated nodes) endow UiO-66 with outstanding water stability [87,88]. Typical Brunauer–Emmett–Teller (BET) surface areas range from 1100 to 1250 m^2^ g^−1^ with a pore volume of ~0.77 cm^3^ g^−1^, making UiO-66 a benchmark metal–organic framework (MOF) widely recognized for its exceptional performance in gas storage, catalysis, water purification, and membrane-based separation, as detailed in the Supplementary Information.

Due to high specific surface area, high porosity, adjustable structure, and surface functionality, UiO-66 have been widely used in catalyst [89,90,91], CO_2_ separation [92,93], hydrogen storage [94,95,96], Ammonia storage [97], drug delivery [98], gas sensing [99,100], and adsorption of pollutants [101,102,103].

**Figure 7:**
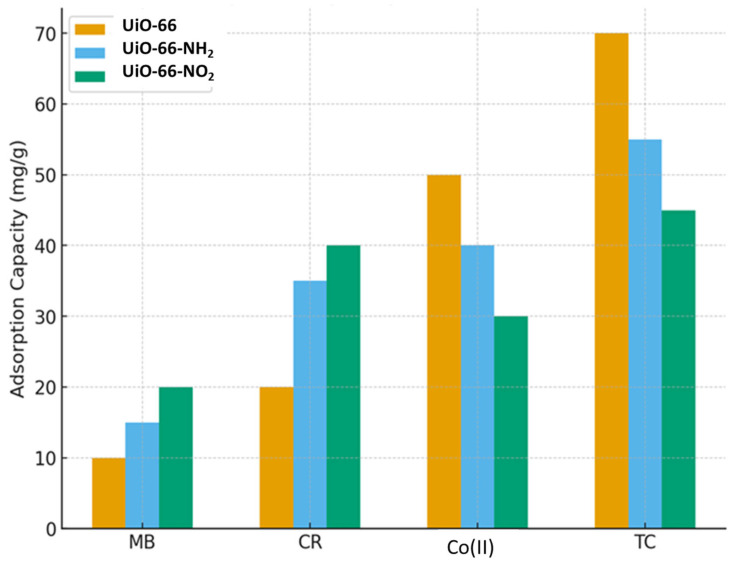
Comparative Adsorption Performance of UiO-66 and Its Functionalized Derivatives (UiO-66-NH_2_ and UiO-66-NO_2_) Toward Congo Red, Methylene Blue, Tetracycline, and Co(II) Ions.

The adsorption performance of UiO-66 and its functionalized derivatives (UiO-66-NH_2_ and UiO-66-NO_2_) toward different pollutants demonstrates clear differences in selectivity and capacity as shown in Figure 7. UiO-66 shows the highest adsorption capacity for tetracycline (70 mg/g) and cobalt ions, indicating its strong affinity toward pharmaceutical contaminants and heavy metal ions. In contrast, UiO-66-NH_2_ performs best with Congo Red, suggesting that amino groups enhance dye adsorption through possible hydrogen bonding or electrostatic interactions. UiO-66-NO_2_, while exhibiting generally moderate adsorption capacities, shows relatively strong uptake for Congo Red and methylene blue compared to its performance with cobalt and tetracycline. Overall, the results highlight that functionalization strongly influences the adsorption selectivity of UiO-66-based materials, with the parent UiO-66 favoring inorganic and pharmaceutical pollutants, while amino- and nitro-functionalized variants improve adsorption of organic dyes [104].

## 5. MOFs for Treatment of Arsenic

A variety of MOFs have been investigated for their potential in arsenic removal as listed in Table 2. Number of studies have focused on MOFs such as Fe-BTC [105], ZIF-8 [106], MOF-74 [107], MIL-100 [108], and so on.

Zhu et al. (2012) [105] pioneered the application of MOFs in arsenic adsorption by synthesizing Fe-BTC. They achieved this by dissolving FeCl_3_·6H_2_O and 1,3,5-benzenetricarboxylic acid (BTC) with a 1:1 molar ratio in 5 mL of N,N′-dimethylformamide (DMF), resulting in a maximum As(V) adsorption capacity of 12.3 mg g^−1^ at pH 4. Zr-based MOF composites have emerged as promising candidates for both As(III) and As(V) removal from water due to their outstanding adsorption capacities. UiO-66, Nu-1000 (Northwestern Univ.-1000) [116], and MOF-808 [117] are prominent examples utilizing Zr as the metal nodes. Li et al. (2015) [113] synthesized MOF-808 using Zr and BTC, achieving an impressive adsorption capacity of As(V) at 24.83 mg g^−1^. Table 2 introduces some of the MOFs applied for arsenic adsorption.

UiO-66, renowned for its high connectivity and robust zirconium-oxygen bonds, holds promise for water treatment applications. Consequently, it demonstrates stability even in acidic or slightly alkaline environments, making it an attractive choice for arsenic removal. Numerous researchers have endeavored to harness its potential for water treatment purposes.

**Figure 8:**
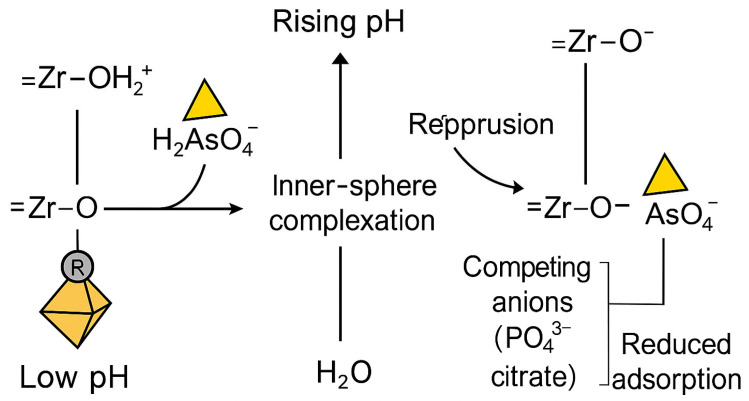
H-dependent inner-sphere complexation of arsenate on UiO-66 Zr nodes. Left: at low pH, Zr–OH sites are protonated (Zr–OH_2_^+^), promoting ligand-exchange with H_2_AsO_4_^−^ to form inner-sphere Zr–O–As linkages with release of H_2_O (bidentate/bridging complexes on Zr_6_ nodes). Right: As pH rises, Zr–OH deprotonates to Zr–O^−^, decreasing electrostatic attraction and discouraging dehydration/condensation; adsorption is further suppressed by competing anions (e.g., PO_4_^3−^, SiO_4_^4−^).

The adsorption behavior of arsenate (As(V)) on UiO-66 and its functionalized derivatives is summarized in Table 3. This behavior is primarily governed by pH-dependent equilibria involving both arsenate speciation and the protonation–deprotonation state of Zr–OH active sites, as illustrated in Figure 8. Under varying pH conditions, the surface charge of UiO-66 frameworks and the ionic form of arsenate collectively determine the strength and mechanism of adsorption. At lower pH, electrostatic attraction between positively charged Zr–OH_2_^+^ sites and negatively charged arsenate species enhances uptake, whereas at higher pH, increased deprotonation of the surface groups weakens this interaction, leading to reduced adsorption capacity.

The apparent discrepancies among studies reporting varying optimal pH values (e.g., Wang et al., 2015) [123] can be rationalized by considering the underlying chemical mechanisms of adsorption. At low pH, the surface of UiO-66 is dominated by protonated Zr–OH_2_^+^ groups, imparting a positive charge that promotes the electrostatic attraction of anionic arsenate species (mainly H_2_AsO_4_^−^). This leads to enhanced adsorption capacities, with Wang et al. (2015) reporting up to 303.4 mg g^−1^ at pH 2. As the pH increases, deprotonation of the surface hydroxyls to Zr–O^−^ reduces the positive surface charge, diminishing electrostatic interactions and decreasing arsenate uptake [18]. Beyond electrostatics, adsorption is primarily driven by inner-sphere complexation between arsenate oxyanions and Zr–OH groups on the UiO-66 nodes. Spectroscopic evidence, including FTIR, XPS, and EXAFS analyses, confirms the formation of bidentate binuclear As–O–Zr linkages) [125] observed the disappearance of As–OH vibrations and emergence of As–O–Zr stretching bands at 830–860 cm^−1^ in FTIR spectra, indicating ligand exchange between arsenate and hydroxyl groups. Chen et al. (2019) [87] similarly reported As 2*p* and O 1*s* binding energy shifts, supporting the formation of covalent Zr–O–As bonds.

This ligand-exchange process proceeds through a dehydration–condensation reaction:

≡Zr–OH_2_^+^ + H_2_AsO_4_^−^ → ≡Zr–O–AsO_3_H + H_2_O

Protonation of Zr–OH sites facilitate this substitution, while at higher pH, de-protonation and repulsive interactions hinder the reaction. Despite this, UiO-66 retains 50–70% of its adsorption capacity under neutral conditions (pH 6–8), relevant for natural waters.

In real groundwater matrices, competing anions such as phosphate (PO_4_^3−^), silicate (SiO_4_^4−^), and natural organic matter (NOM) significantly reduce As(V) uptake by occupying Zr–OH sites or forming outer-sphere complexes, leading to 15–40% efficiency loss [84]. Nevertheless, the strong inner-sphere complexation mechanism grants UiO-66 resilience to moderate competition, especially when defects and functional groups (–NH_2_, –SH) enhance site accessibility and reactivity.

According to literature, UiO-66 exhibits a zero-point charge at pH 4.3 [126], indicating that the adsorbent surface carries a net positive charge below this pH and a net negative charge above it. In experiments by Assaad et al. (2020), at pH = 2, arsenate exists in its neutral form (H_3_AsO_4_), suggesting low adsorption due to absence of charge [126]. However, the maximum adsorption capacity was achieved at this pH, suggesting that electrostatic attraction was not the sole mechanism governing arsenate uptake. Instead, specific coordination interactions also play a crucial role. Arsenate ions are believed to bind to the MOF surface through dual coordination mechanisms analogous to acid–base interactions, wherein acidic conditions promote the dissociation of protons (H^+^) from H_3_AsO_4_, facilitating stronger binding with surface hydroxyl (–OH) groups on the MOF. In other studies, UiO-66 exhibited enhanced arsenic removal efficiency at moderately alkaline pH values, with performance decreasing sharply beyond pH > 11, likely due to the deprotonation of active sites and reduced electrostatic attraction [18,115].

The influence of acidic and basic environments on a series of zirconium-based metal–organic frameworks (Zr-MOFs) was systematically examined by Kandiah et al. (2010) [127]. Their findings revealed that all evaluated MOFs retained full crystallinity when exposed to aqueous and acidic conditions (HCl, pH = 1). However, under strongly basic conditions (NaOH, pH = 14), only UiO-66–NO_2_ maintained its structural integrity, while UiO-66 and UiO-66–Br exhibited minor alterations in their crystal frameworks. In contrast, UiO-66–NH_2_ underwent complete structural decomposition within two hours, highlighting its reduced chemical stability in highly alkaline media.

The structural properties of UiO-66—particularly pore size, surface area, and the presence of structural defects play a decisive role in determining its arsenic adsorption performance. While pristine UiO-66 exhibits a high theoretical surface area (~1180 m^2^ g^−1^) and microporous cages (tetrahedral ≈ 8 Å, octahedral ≈ 11 Å, interconnected by ~6 Å triangular windows), these narrow pore openings often limit accessibility to hydrated arsenate ions (≈ 7.7 Å). As a result, a significant portion of the internal porosity remains underutilized for adsorption [119]. This size exclusion effect constrains both the equilibrium uptake and the adsorption rate, particularly for larger hydrated species such as H_2_AsO_4_^−^ and HAsO_4_^2−^.

To overcome this diffusion barrier, defect engineering and modulator-assisted synthesis have emerged as powerful strategies to introduce mesoporosity and coordinatively unsaturated Zr sites, thereby improving both capacity and kinetics. Modulators such as HCl, trifluoroacetic acid (TFA), and acetic acid (AA) selectively replace linkers during crystal growth, generating missing-linker and missing-cluster defects that expand pore channels and expose Zr–OH groups for stronger As–O–Zr bonding [126]. The resulting mesopores (2–50 nm) significantly accelerate intraparticle diffusion and enhance adsorption kinetics, shortening equilibrium times from several hours to under one hour in some systems [118,128].

Defected or modulated UiO-66 samples often exhibit surface areas exceeding 1000 m^2^ g^−1^, compared with ~400–800 m^2^ g^−1^ for unmodified UiO-66-NH_2_ (Table 3). For example, UiO-66-TFA/AA achieved a surface area of 1690 m^2^ g^−1^ and an exceptional As(V) capacity of 365 mg g^−1^ [126], whereas defect-rich UiO-66 (DU) prepared by Somjit et al. (2022b) [129] reached 204 mg g^−1^ for As(III) [129]. The lattice-defected UiO-66 (t-ZrO_2_) synthesized by Qu et al. (2022) [128] demonstrated enhanced dual As(III)/As(V) removal (352.1 mg g^−1^ and 147.5 mg g^−1^, respectively), confirming the beneficial role of defective sites in improving both adsorption energy and reaction kinetics.

Spectroscopic evidence (FTIR, XPS, and EXAFS) suggests that these structural defects not only increase accessibility but also create coordinatively unsaturated Zr sites capable of forming stronger inner-sphere complexes with arsenate or arsenite species. Moreover, defect-induced mesoporosity facilitates the formation of local microenvironments that favor the dehydration of arsenate ions and subsequent ligand exchange at Zr–OH nodes.

However, a balance must be maintained—excessive defect density may compromise structural integrity and hydrolytic stability, leading to partial collapse or Zr leaching under aqueous conditions. Therefore, combining controlled defect engineering with functionalized ligands (e.g., –NH_2_, –SH, or –COOH) and magnetic or carbon supports offers a rational design route. This hybrid approach enhances arsenic uptake, accelerates adsorption kinetics, and preserves mechanical durability during cyclic operation.

In summary, increasing mesoporosity and defect density within UiO-66 frameworks directly correlates with higher arsenic uptake capacities and faster kinetics by improving mass transfer and active site exposure. Future work should aim to quantify the optimal defect concentration and assess long-term stability and regeneration under realistic water chemistries containing competing ions such as phosphate, silicate, and natural organic matter.

Research using the X-ray Photoelectron Spectroscopy (XPS) and Fourier Transform Infrared Spectroscopy (FTIR) technique indicates that Zr-O bonding is important for As removal from UiO-66 [115]. He et al. (2019) reported that, through X-ray Absorption Near Edge Structure spectroscopy (XANES), both As(III) and As(V) were anchored on UiO-66 without a change in oxidation state [115]. Extended X-ray absorption fine structure spectroscopy (EXAFS) analysis further revealed that arsenate [As(V)] primarily forms bidentate mononuclear complexes, whereas arsenite [As(III)] tends to establish bidentate binuclear coordination with the hexanuclear Zr clusters of UiO-66. These findings highlight the pivotal role of Zr–O active sites in governing arsenic binding mechanisms. Overall, the study demonstrates that zirconium-based metal–organic frameworks (MOFs), owing to their exceptional structural stability and low toxicity, represent promising candidates for efficient arsenic sequestration from aqueous environments.

## 6. Strategies for Enhancement Arsenic Removal of UiO-66

The functionalization of UiO-66 linkers and nodes provides an effective strategy to tailor interfacial chemistry for arsenic uptake while influencing hydrolytic stability, oxidative durability, and regeneration performance.

Amino functionalization (–NH_2_) enhances the adsorption of As(V) primarily through hydrogen bonding and inner-sphere complexation at Zr–OH nodes. Protonation to –NH_3_^+^ under acidic conditions favors electrostatic attraction toward H_2_AsO_4_^−^, accelerating adsorption kinetics and increasing capacity [119,130]. However, at higher pH values, deprotonation diminishes these interactions and may expose coordinatively unsaturated Zr sites, potentially affecting long-term framework stability. UiO-66–NH_2_ remains structurally stable within pH 4–10 [115], but strong alkaline environments (pH > 12) can induce partial hydrolysis [120].

Thiolated UiO-66 (–SH) offers strong affinity for both As(III) and As(V) via soft–soft Lewis acid–base interactions [121,122]. Nevertheless, the oxidation of –SH to disulfides (–S–S–) under aerobic or alkaline regeneration reduces available active sites. Strategies such as conducting regeneration under oxygen-limited conditions, adding mild antioxidants, or integrating Fe_3_O_4_ supports can mitigate oxidation and sustain functionality.

Electron-withdrawing groups like –NO_2_ enhance alkaline resistance by strengthening Zr–O coordination [127], while chelating linkers such as EDTA introduce multiple donor atoms for polyvalent metal binding [124]. However, bulky ligands may hinder mass transport within pores, highlighting a trade-off between functional density and diffusion efficiency.

Regeneration performance remains a key metric for real-world applicability as summarized in Table 4. Common desorption reagents include NaOH (0.1–0.5 M), NaCl, and NaHCO_3_. Most functionalized UiO-66 variants retain 80–95% of their initial adsorption capacity after 4–6 cycles, although minor Zr or Fe leaching (1–3%) has been reported. Structural analyses confirm minimal degradation: PXRD patterns retain the primary reflections of UiO-66 (2θ ≈ 7.4°, 8.5°, 25.8°) with <10% intensity loss, suggesting only partial pore blockage by residual arsenate species [115,120]. N_2_ sorption isotherms at 77 K preserve a Type I profile with a slight H4 hysteresis loop, confirming stable micro-mesoporosity. UiO-66–NH_2_ maintains 85–93% of its initial BET area, whereas UiO-66–SH exhibits ~20% loss due to –SH oxidation [125]. Defect-engineered UiO-66 and modulator-assisted variants such as UiO-66–TFA/AA exhibit excellent stability, retaining 90–95% BET area and pore volume after cycling [126,128]. FTIR and XPS studies confirm persistence of As–O–Zr linkages and ligand integrity, validating that the primary adsorption mechanism remains active upon regeneration.

Ligand type dictates regeneration tolerance: amino-functionalized UiO-66 is more compatible with alkaline eluents, while thiolated systems perform better under neutral or mildly basic conditions. Future research should quantify correlations between ligand chemistry, eluent pH, and post-cycle capacity retention under realistic water matrices containing phosphate, silicate, and natural organic matter (NOM)—all known to compete with arsenate.

Defect and modulator engineering synergize with functionalization to improve uptake and kinetics. Controlled defect creation through modulators such as HCl, trifluoroacetic acid (TFA), or acetic acid introduces mesoporosity and coordinatively unsaturated Zr sites. These modifications increase accessibility of Zr–OH nodes and enhance intraparticle diffusion. For instance, UiO-66–TFA/AA demonstrates arsenic adsorption capacities up to 365 mg g^−1^ with equilibrium reached within 1 h [109]. However, excessive defect density can compromise mechanical stability and water tolerance. Therefore, optimizing defect density, ligand functionality, and framework stability is essential for achieving high capacity, fast kinetics, and multi-cycle durability in sustainable arsenic remediation.

The addition of modulators in MOF synthesis plays a crucial role in controlling crystal growth, enhancing structural stability, and tuning defect concentration. Modulators typically consist of monocarboxylic acids (R–COOH) such as acetic acid (AA), formic acid (FA), benzoic acid (BA), and trifluoroacetic acid (TFA), which compete with the organic linker for coordination sites at the Zr_6_ nodes [80]. This competition influences nucleation kinetics, particle size, and the degree of missing-linker or missing-cluster defects, which ultimately modulate the material’s adsorption behavior and mass transfer characteristics. Defect creation in UiO-66 can be achieved either through intrinsic linker deficiency or via modulator-assisted synthesis. Direct defect formation occurs when stoichiometric imbalance between linker and metal source induces missing linkers or clusters, exposing coordinatively unsaturated Zr–OH sites that serve as Lewis acid centers for arsenate binding. Such sites have been quantified using thermogravimetric analysis (TGA) and solution ^1^H NMR of digested MOF samples, revealing missing-linker concentrations of 1–3 per node in modulated UiO-66 variants [132]. Diffuse Reflectance Infrared Fourier Transform Spectroscopy (DRIFTS) further confirms enhanced Zr–OH stretching bands, correlating with the number of unsaturated sites and thus higher adsorption capacities. In contrast, acid/base co-modulation—using agents such as HCl and triethylamine (TEA)—influences both defect formation and crystal morphology by controlling deprotonation kinetics during nucleation. Shan et al. (2018) demonstrated that TEA-assisted deprotonation promotes partial ligand substitution and generates missing-cluster defects, yielding intergrown UiO-66 films with enhanced mesoporosity and improved accessibility of internal adsorption sites [133]. Zhao et al. (2017) later expanded this approach, showing that combining acetic acid and TEA enabled precise control over crystal size and uniformity [134].

Acidic modulators such as HCl or TFA promote the generation of coordinatively unsaturated Zr sites by protonating linkers, thereby weakening Zr–O bonds and facilitating controlled defect formation. Chang et al. (2019) [118] used HCl as a modulator for UiO-66–NH_2_, which increased arsenate adsorption capacity to 161.3 mg g^−1^ at pH 7—significantly higher than the unmodified counterpart. Similarly, Assaad et al. (2020) synthesized a series of UiO-66 materials using varying ratios of AA and TFA, achieving As(V) adsorption capacities from 89.3 to 365.4 mg g^−1^ [126]. These enhancements are attributed to increased mesoporosity and the formation of free Lewis acid sites induced by trifluoroacetate substitution.

Basic co-modulation, on the other hand, affects deprotonation rates and mass transport efficiency. TEA-assisted nucleation typically leads to smaller, more uniform crystallites and higher external surface areas, accelerating adsorption kinetics but occasionally at the expense of framework density. Vermoortele et al. (2013) showed that acid–base pairs (HCl–TFA) produce frameworks with high defect densities yet maintain stability due to controlled charge compensation at the Zr nodes [135]. Qu et al. (2022) employed sodium acetate as a mild modulator, generating lattice-defected UiO-66 with a maximum As(III) capacity of 352.1 mg g^−1^ and improved pore volume (0.44 cm^3^ g^−1^) [128]. Such results suggest that both acidity and diffusion play synergistic roles in optimizing As uptake.

Defect-engineered and hydroxylated variants of UiO-66 also show improved mass transfer and surface reactivity. Somjit et al. (2022b) [129] reported that hydroxyl substitution of linkers increased pore volume from 0.34 to 0.44 cm^3^ g^−1^ and raised As(III) adsorption capacity from 78.23 to 204 mg g^−1^, even at pH values as low as 1–4 [129]. The increase in mesoporosity enhances intraparticle diffusion and accessibility of Zr–OH sites, thus accelerating equilibrium uptake. While defect introduction improves kinetics and capacity, excessive defect densities can compromise mechanical integrity and water stability, emphasizing the need for optimized defect balance.

Characterization techniques such as PXRD, N_2_ sorption, TGA, solution NMR, and DRIFTS collectively confirm that moderate modulator-induced defect formation enhances arsenic adsorption primarily through improved site availability and mass transfer rather than acidity alone. Future work should focus on quantifying defect densities, establishing correlations with adsorption kinetics, and integrating in situ spectroscopic monitoring to elucidate mass transport and coordination mechanisms under realistic aqueous conditions.

UiO-66 analogs advance arsenic water treatment by combining high stability with tunable chemistry. Functional modifications (–NH_2_, –SH) enhance As(V)/As(III) binding through hydrogen bonding and inner-sphere complexation, while defect engineering via modulators (AA, TFA, HCl) increases unsaturated Zr–OH sites and mesoporosity, improving kinetics and capacity (Table 5). Node substitution (Zr^4+^→Hf^4+^, Ce^4+^, Ti^4+^) further tunes acidity and redox activity for catalytic detoxification. These analogs maintain 80–95% capacity over multiple regeneration cycles with minimal leaching, as verified by PXRD and N_2_ sorption. Environmentally benign, water-based synthesis routes make UiO-66 analogs promising, scalable adsorbents for sustainable arsenic remediation in complex water systems.

**Figure 9:**
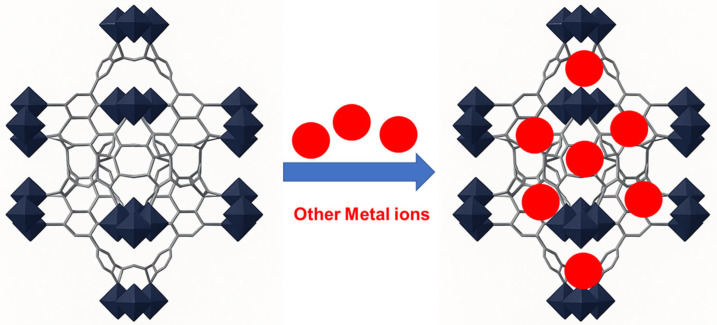
Introducing different metal ions (red) into MOFs (black), to enhance its adsorption properties.

Therefore, metal doping, which involves introducing different metal ions at specific locations within the structure of MOFs such as UiO-66, can enhance its adsorption properties (adsorption capacity and/or selectivity) as shown in Figure 9. Metal doping introduces additional active sites on the surface of UiO-66, thereby increasing its adsorption capacity. Furthermore, doped metal ions can form specific bonding configurations within the structure of UiO-66, leading to improved interactions with pollutants. The integration of different metal ions within a MOF structure can generate synergistic interactions that improve the reactivity and adsorption performance toward arsenic species [136].

An alternative and particularly elegant route to obtain magnetic nanoparticle (MNP)@MOF composites is the so-called “ship-in-a-bottle” strategy, which relies on the in situ formation of MNPs within a pre-synthesized metal–organic framework (MOF) [79]. In this approach, the MOF serves as a confined reaction vessel or nanoreactor that directs the nucleation and growth of MNPs, thereby allowing for better control over particle dispersion and size distribution.

**Figure 10:**
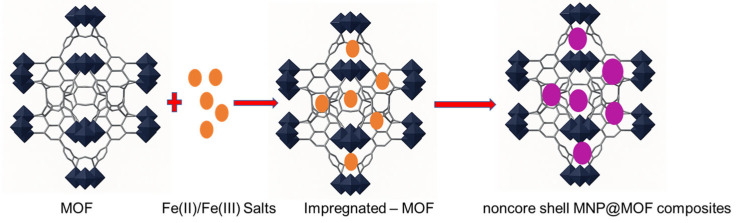
Schematic representation of the in-situ formation of magnetic nanoparticles within a pre-synthesized MOF, illustrating the “ship-in-a-bottle” synthesis strategy for non–core–shell MNP@MOF composites.

The synthesis generally proceeds through two main stages (Figure 10). First, iron ions or suitable MNP precursors are introduced into the preformed MOF, most commonly via chemical vapor infiltration, solution impregnation, or incipient wetness infiltration methods [80]. These techniques facilitate the diffusion and immobilization of metal precursors within the MOF’s porous structure. In the second step, the incorporated precursors undergo chemical transformation. Typically, through phase or topotactic transitions, dehydration, or reduction processes, to yield the corresponding iron oxide nanoparticles [81]. The transformation step can be thermally or chemically induced, and the precise conditions strongly influence the resulting nanoparticle phase, crystallinity, and size (see Figure 8).

The spatial distribution of the generated MNPs depends largely on the interplay between the MOF’s pore size and the kinetics of nucleation and growth. In many cases, nanoparticles are located on the external surface or partially embedded within the pore system. When the nanoparticle size approaches or exceeds the accessible pore dimensions, partial framework degradation or defect formation may occur, leading to the creation of mesoporosity or partial collapse of the structure. This defect formation represents a delicate equilibrium to control—excessive disruption can compromise the MOF’s crystallinity and stability.

Conversely, under optimized conditions, the MNPs can be uniformly dispersed throughout the MOF [80]. In such cases, the porous network effectively stabilizes the nanoparticles, providing physical confinement and chemical protection against aggregation, oxidation, and leaching. This structural synergy enhances the material’s long-term stability and magnetic functionality, making the MNP@MOF composite particularly attractive for applications in catalysis, magnetic separation, and environmental remediation.

**Figure 11:**
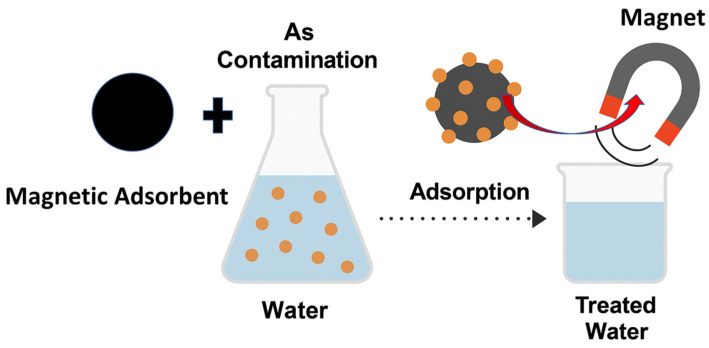
Schematic illustration of arsenic removal from water using magnetic adsorbents.

Arsenic-contaminated water is effectively treated through the adsorption of arsenic ions onto magnetic nanoparticle (MNP)-based composite materials, followed by their rapid separation from the aqueous phase using an external magnetic field, as illustrated in Figure 11. In this process, the MNPs or MNP@MOF composites serve as efficient adsorbents owing to their high surface area, tunable surface chemistry, and strong magnetic responsiveness, which facilitate both high arsenic uptake and easy recovery. The application of a magnetic field allows for the swift isolation of the spent adsorbent without requiring filtration or centrifugation, thereby simplifying the water purification process and minimizing secondary waste.

**Figure 12:**
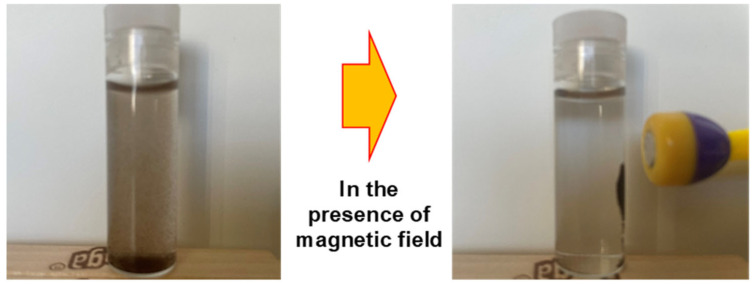
Illustration of easy recovery of M-UiO after arsenic adsorption.

Figure 12 presents the conceptual design developed in our laboratory, demonstrating the practical use of this method, from the initial contact between arsenic-laden water and the magnetic adsorbent to the subsequent magnetic separation step, resulting in clean, arsenic-free water ready for safe use or discharge.

Feng et al. (2017) synthesized a γ-Fe_2_O_3_@ZrO_2_ composite, consisting of γ-Fe_2_O_3_ magnetic cores encapsulated within a ZrO_2_ shell, to enhance arsenic removal efficiency [137]. The material exhibited maximum adsorption capacities of 62.2 mg g^−1^ for As(III) and 18.3 mg/g for As(V) at pH 9, demonstrating its dual functionality as both an adsorbent and magnetic separable medium. Although not based on UiO-66, Yang and Yin (2017) reported outstanding arsenic removal performance using a CoFe_2_O_4_@MIL-100(Fe) composite [138]. This hybrid material achieved adsorption capacities of 114.8 mg g^−1^ for As(V) and 143.6 mg g^−1^ for As(III), with near-complete removal (~100%) observed across a wide pH range (2–12). The remarkable performance was attributed to the abundant surface hydroxyl (–OH) groups and the lower point of zero charge (pHpzc) resulting from the synergistic interaction between CoFe_2_O_4_ and MIL-100(Fe) components. In a more recent study, Pervez et al. (2022) developed cerium-based metal–organic frameworks (Ce-MOFs), including Ce-MOF-66 and Ce-MOF-808, as analogs to the UiO-66 structure [139]. Substituting Zr^4+^ with Ce^4+^ in the UiO-66 lattice significantly enhanced arsenic affinity, yielding a maximum adsorption capacity of 355.7 mg g^−1^ for As(V) in Ce-MOF-66. Moreover, Ce-MOF-808, incorporating benzene-1,3,5-tricarboxylate (BTC) linkers instead of benzene-1,4-dicarboxylate (BDC) demonstrated adsorption capacities of 217.79 mg g^−1^ for As(V) and 402.09 mg g^−1^ for As(III), underscoring the potential of Ce-based MOFs for efficient arsenic remediation.

The UiO-66@supporter structures using various supports such as ZVI, Magnetite, Porous Graphic Carbon (PGC), and graphene oxide (GO), among others, can bring excellent effects in arsenic adsorption. Using magnetic supports like Fe_3_O_4_@UiO-66 allows for the easy recovery of the UiO-66 adsorbent, enhancing its functionality as a recyclable adsorbent. Additionally, magnetic supports can enhance the adsorption capacity and selectivity of UiO-66. PGC or GO as porous supports increases the surface area of UiO-66, introducing new adsorption sites to enhance the adsorption capacity of arsenic ions. Moreover, these supports can improve the mechanical strength of UiO-66, enhancing the stability of the adsorbent. Liu et al. (2019) coupled nanometer zero-valent iron (NZVI) with UiO-66 to enhance the adsorption efficiency of As(III) [140]. Their experiments demonstrated that the NZVI@UiO-66 composite exhibited robust anti-interference capabilities against co-existing ions (such as Ca^2+^, Mn^2+^, Cu^2+^, H_2_PO_4_^−^, and SO_4_^2−^) and organic acids (including oxalic acid and citric acid). This composite facilitated the effective removal of As(III) and its oxidized product, As(V), achieving an impressive adsorption capacity of 360.6 mg As per gram of NZVI through a combination of chemical oxidation and physical adsorption mechanisms. Moreover, NZVI@UiO-66 demonstrated rapid and efficient removal of As(III) within a short time frame (on the scale of minutes) across a wide pH range (from 3.0 to 12.0) and concentration range (from 20 to 100 mg/L).

Magnetic properties not only enhance catalytic activity but also facilitate easy recovery of the adsorbent using magnets. The adsorption capacity of magnetic UiO-66 for arsenic rivals that of most MOFs-containing adsorbents, yet its magnetic properties enable easy separation of the composite from the aqueous solution (Figure 12). Many researchers have utilized magnetite nanoparticles (MNP) in MOFs to impart magnetic properties, employing both non-core–shell and core–shell types [141]. Core–shell MNP@MOFs composites offer diverse designability and enhanced selectivity [142]. Chen et al. (2019) demonstrated significantly enhanced catalytic activity through Layer-by-Layer Fabrication of Core–Shell Fe_3_O_4_@UiO-66-NH_2_, which was 36 times higher than UiO-66-NH_2_ alone [143].

Qi et al. (2019) synthesized Fe_3_O_4_@TA@UiO-66 magnetic core–shell microspheres using tannic acid, which served as a supportive agent [144]. Tannic acid played a beneficial role in preventing spontaneous aggregation and improving the dispersibility of Fe_3_O_4_ by facilitating the self-assembly of active phenol hydroxyl groups. The specific surface area of Fe_3_O_4_@TA@UiO-66 microspheres was measured at 130.3 m^2^/g. These microspheres exhibited removal efficiency for As(III) at 97.8 mg/g due to their unique magnetism, thereby demonstrating promising practical applications. Huo et al. (2019) synthesized Fe_3_O_4_@UiO-66 magnetic nanoparticles using a two-step solvothermal method with the addition of acetic acid [145]. The specific surface area of Fe_3_O_4_@UiO-66 was reported as 124.8 m^2^/g. This composite demonstrated a removal capacity of 73.2 mg/g for As(V). Interestingly, Fe_3_O_4_@UiO-66 exhibited faster kinetics compared to UiO-66 alone, with the rate coefficients following the order: Fe_3_O_4_ < UiO-66 < Fe_3_O_4_@UiO-66. It is noteworthy that Fe_3_O_4_@UiO-66 exhibited synergistic performance, potentially attributed to strong coordination between Zr ions and arsenate, as well as the unique core–shell structure. The latter could expose more active sites due to its high specific surface area, leading to enhanced arsenate removal efficiency.

Ahmadipouya et al. (2021) accelerated the removal rate of underwater organic dyes using Magnetic Fe_3_O_4_@UiO-66 nanocomposite [146]. Ahmadijokani et al. (2021) also using Fe_3_O_4_@PAA@UiO-66-NH_2_ for selective adsorption of the anti-cancer Quercetin (QCT) drug. PAA is poly (acrylic acid) [147]. Regeneration and reusability experiments revealed that the developed adsorbent maintained excellent structural stability and consistent adsorption performance for QCT even after ten consecutive adsorption–desorption cycles, demonstrating its durability and practical applicability for repeated use.

The incorporation of magnetic nanoparticles such as Fe_3_O_4_ or γ-Fe_2_O_3_ into UiO-66 frameworks enables efficient magnetic recovery and reusability, which are essential for practical water treatment applications (Table 6). Magnetically responsive UiO-66 composites (e.g., Fe_3_O_4_@UiO-66, UiO-66–Fe) allow rapid magnetic separation within 30–60 s under an external magnetic field, thereby minimizing secondary waste generation and eliminating the need for filtration or centrifugation [118,125]. Such facile recovery significantly enhances operational sustainability. Repeated adsorption–desorption cycles, however, may induce partial particle loss and metal ion leaching. Fe leaching usually results from oxidation or partial detachment of Fe_3_O_4_ cores, while Zr leaching can occur due to hydrolysis of metal–ligand bonds under extreme pH. In most reports, Fe leaching remains below 1.5% and Zr below 3% after five cycles [115,129]. UiO-66–NH_2_/Fe_3_O_4_ composites have demonstrated over 90% capacity retention after six regeneration cycles with negligible magnetic weakening, confirming high stability and recyclability [126]. PXRD analyses show preserved crystallinity, and N_2_ sorption results indicate minimal reduction (<10%) in surface area after cycling, validating framework durability.

Leaching and particle loss can be precisely quantified using Inductively Coupled Plasma Optical Emission Spectroscopy (ICP–OES) or Inductively Coupled Plasma Mass Spectrometry (ICP–MS). After each cycle, the supernatant is filtered through a 0.22 μm membrane, acidified with 2% HNO_3_, and analyzed to determine trace Fe, Zr, or As concentrations, typically down to detection limits of ~0.1 μg L^−1^. Complementary solid mass recovery measurements and BET surface area analyses provide insights into physical loss and structural stability. A representative study reported <5% total particle loss after six cycles, stable magnetization (~45 emu g^−1^), and unchanged PXRD profiles, demonstrating robust performance and excellent magnetic recyclability.

Collectively, these findings confirm that UiO-66-based magnetic composites offer durable performance, minimal metal leaching, and strong magnetic recovery efficiency. Continued optimization of linker chemistry, core–shell design, and regeneration conditions will further enhance their applicability for large-scale, environmentally safe arsenic remediation.

Pandi et al. (2020) synthesized UiO-66@PGC by employing porous graphic carbon [114]. The maximum adsorption capacities of As(III) and As(V) were increased from 110.47 mg/g to 185.38 mg/g and 132.18 mg/g to 201.02 mg/g, respectively. Singh et al. (2022) synthesized UiO-66-NDC by incorporating graphene oxide (GO) into zirconium-based MOFs [148]. UiO-66-NDC features a three-dimensional structure comprising one octahedral center hole cage, eight tetrahedral corner cages of secondary building units Zr_6_O_4_(OH)_4_, and twelve bridge ligands 1,4-NDC. It possesses a notable number of coordinatively unsaturated Zr^4+^ sites and a robust ZrO bond, facilitating adsorption and mass transfer. These materials exhibit resistance to hydroxide ions and protons and remain stable across various wastewater compositions and pH levels.

**Table 6:** Some list of different UiO-66 adapted metal doping or UiO-66@Supporter used for arsenic adsorption from water.

Adsorbents	Pollutants	BET Surface Area (m^2^ g^−1^)	$q_{max}$ (mg g^−1^)	References
γ-Fe_2_O_3_@ZrO_2_	As(III)/As(V)		62.2/18.3	Feng et al. (2017) [137]
CeO_2_/Fe_3_O_4_@UiO-66 & CeO_2_/Fe_3_O_4_@UiO-66-(SH)_2_	various metal	597, 539	Real River As 86~99%	Boix et al. (2020) [149]
Ce-MOF-66	As(III)/As(V)		5.51/355.66	Pervez et al. (2022) [139]
Ce-MOF-808	As(III)/As(V)		402.09/217.79	Pervez et al. (2022) [139]
S-CuLa@UiO-66	As(III)		171	Jiang et al. (2021) [150]
UiO-66 (Fe/Zr)	As(III)/As(V)	498.33	102/204	Guo et al. (2023) [151]
ZrFc-MOF/ ZrFc-MOF/PMS ^1)^	As(S)		59.59/111.34	Li et al. (2023) [152]
NZVI ^2)^@UiO-66	As(III)		360.6	Liu et al. (2019) [140]
Fe_3_O_4_@UiO-66-NH_2_		76.3	36 times higher than the UiO-66-NH_2_	Chen et al. (2019) [143]
Fe_3_O_4_@UiO-66	As(V)	124.8	73.2	Huo et al. (2019) [145]
Fe_3_O_4_@TA ^3)^@UiO-66	Sb(III)/As(III)	130.3	49.51/97.82	Qi et al. (2019) [144]
BSMM ^4)^	Sb(III)	55	18.43	Zhu et al. (2021) [153]
UiO-66@PGC ^5)^	As(III)/As(V)	1312.37	185.38/201.02	Pandi et al. (2020) [114]
UiO-66-NDC ^6)^/GO ^7)^	As(V)	229.78	147.06	Singh et al. (2022) [148]

^1)^ PMS: peroxymonosulfate, ^2)^ NZVI: nano ZVI, ^3)^ TA: Tannic acid, ^4)^ BSMM: Biochar Supported Magnetics MOFs, ^5)^ PGC: Porous Graphic Carbon, ^6)^ NDC: 1,4-naphtalenedicarboxylate, ^7)^ GO: graohene oxide. (Based on Langmuir Model).

The commercial application of MOFs raises questions about their suitability as “environment-friendly materials” for a sustainable future. Environmental challenges associated with MOFs stem from the complexes and solvents used in their synthesis [154], which are not limited to UiO-66 but are relevant to all MOFs. Several researchers have raised concerns about the potential toxicity of MOFs [132,155,156]. Kumar et al. (2019) suggested that the release of metal ions and linker molecules from certain MOFs could be a cause of their toxic effects [157]. In other words, metal ions, linkers, modulators, and solvents used in the synthesis process of MOFs may leach into water during application. To address these concerns, methods are needed to minimize or eliminate the use of hazardous and non-renewable solvents such as dimethylformamide (DMF), dimethylformamide (DEF), methanol, ethanol, and acetonitrile, which are commonly used in solvothermal processes for MOF synthesis. Alternative processes include water-based MOF synthesis, mechanochemical MOF synthesis, and the use of supercritical fluids and ionic liquids as solvents to replace conventional ones [154]. In response to these challenges, many researchers have introduced the concept of “green synthesis” [158,159]. Despite its toxicity, N,N-dimethylformamide (DMF) is still considered the most efficient solvent for obtaining high-quality UiO-66. Venturi et al. (2020) conducted a survey of approximately 40 different solvents used in laboratory-scale synthesis of high-quality UiO-66 crystals, identifying γ-valerolactone (GVL) as one of the most promising solvents for replacing DMF [160]. An et al. (2023) demonstrated the seed-aided synthesis of eleven MOFs with diverse compositions and structures using pure water as the solvent [159]. This approach addresses challenges associated with switching from organic solvents to water, including poor solubility of organic linkers, slow reaction kinetics, and the formation of polymorphic products.

## 7. Green Synthesis of UiO-66: Transitioning from DMF to Sustainable Solvents

The replacement of N,N-dimethylformamide (DMF) in the synthesis of UiO-66 is a critical step toward safer and more sustainable large-scale production of metal–organic frameworks (MOFs). DMF, although widely used due to its excellent solvation properties and ability to promote crystalline growth, presents significant challenges: it is toxic, volatile, difficult to recycle, and hazardous for workers during scale-up. Consequently, green solvent alternatives such as γ-valerolactone (GVL) and water-based systems are emerging as promising substitutes [85,126,132,133].

GVL, a renewable solvent derived from biomass, provides a lower-toxicity alternative to DMF while maintaining similar polarity and coordination behavior. Studies have demonstrated that GVL-based solvothermal syntheses yield UiO-66 with Brunauer–Emmett–Teller (BET) surface areas of 1000–1400 m^2^ g^−1^—comparable or even superior to DMF-prepared materials—when optimized with modulators such as acetic acid (AA), trifluoroacetic acid (TFA), or hydrochloric acid (HCl). Moreover, GVL facilitates defect engineering through controlled modulator activity, creating hierarchical mesoporosity beneficial for arsenic adsorption and rapid diffusion kinetics.

Water-based or ambient syntheses further minimize environmental burden. While early reports indicated reduced crystallinity and surface area, advances in modulator-assisted aqueous routes now achieve BET areas of 900–1250 m^2^ g^−1^ and well-defined pore structures. Using acid/base co-modulation (AA + TEA or HCl + TFA), water syntheses can generate hierarchical porosity and defect densities comparable to DMF-derived materials. These properties translate into enhanced mass transfer and faster arsenic uptake, often reaching equilibrium within one hour.

When comparing synthetic routes side-by-side, the critical differentiator is not the solvent itself but the interplay between medium polarity, modulator acidity, and mass transport. Acidic modulators promote higher proton activity and deprotonation efficiency of the organic linker, while mesoporosity arising from controlled defect generation improves kinetic accessibility. Both mechanisms are achievable under GVL or aqueous conditions, demonstrating that solvent replacement does not inherently compromise performance.

Future research should focus on developing environmentally benign and cost-effective synthesis routes for MOF-based materials applicable to large-scale arsenic removal from water. In particular, the use of water as a green solvent for the synthesis of MOFs presents a promising and sustainable alternative to conventional solvothermal methods, which often rely on toxic organic solvents and elevated temperatures. The successful fabrication of TAF-CNU-1 in aqueous media at room temperature [161] demonstrates the feasibility of producing highly crystalline and porous frameworks under mild, eco-friendly conditions.

Building upon this concept, future efforts could aim to design and synthesize magnetic or defect-engineered MOFs via water-based synthesis to enhance both adsorption efficiency and recyclability in arsenic remediation applications. Such an approach would not only minimize environmental impact and energy consumption but also facilitate scalable, low-cost production suitable for industrial deployment. Additionally, integrating green synthesis strategies with post-synthetic modification or composite formation (e.g., MNP@MOF hybrids) could further improve selectivity, kinetics, and magnetic separability, paving the way for next-generation adsorbents tailored for sustainable water purification systems.

Comprehensive structural and adsorption benchmarking confirms parity across green and traditional syntheses. Powder X-ray diffraction (PXRD) patterns of UiO-66(GVL) and UiO-66(H_2_O) retain the characteristic reflections of the fcc topology (2θ ≈ 7.4°, 8.5°, and 25.8°), indicating preserved crystallinity. N_2_ sorption isotherms exhibit Type I hysteresis, consistent with microporous–mesoporous networks. Regeneration studies show 85–95% retention of adsorption capacity after five cycles, with less than 3% Zr leaching. These findings highlight those green syntheses maintain the durability, porosity, and arsenic uptake efficiency observed in DMF-based systems.

Together, these results support the transition from DMF to GVL or water-based syntheses as a practical and environmentally responsible pathway for UiO-66 fabrication. Optimizing the solvent–modulator–defect triad offers a clear route to maintaining high capacity (80–300 mg g^−1^), fast kinetics, and excellent structural durability while dramatically lowering ecological and occupational risks.

## 8. Conclusions

Arsenic contamination continues to pose serious health and environmental risks, demanding effective, durable, and scalable treatment technologies. Among the existing methods, adsorption using metal–organic frameworks (MOFs), particularly UiO-66 and its derivatives, has shown outstanding potential due to their high surface area, tunable chemistry, and stability under aqueous conditions. Modified UiO-66 materials such as NH_2_-UiO-66, Fe-doped UiO-66, and Fe_3_O_4_@UiO-66 composites have achieved arsenic adsorption capacities ranging from 80 to 150 mg g^−1^, with optimal performance at pH 4–8 and resilience across moderate ionic strengths. These materials also exhibit 70–95% regeneration efficiency over several cycles with limited Zr or Fe leaching (<3%), underscoring their potential for long-term application.

However, real water matrices introduce complexities not fully captured in laboratory systems. The presence of phosphate, silicate, and natural organic matter (NOM) strongly competes with arsenic for active sites, often suppressing adsorption by 20–50%. Enhancing durability under such conditions—while maintaining easy separation and reusability, remains a key challenge.

Future development of UiO-66-based adsorbents should therefore integrate green, water-based synthesis, controlled defect engineering, and surface functionalization to optimize both selectivity and stability. Combining defective frameworks (to create open metal sites), functional groups (e.g., –NH_2_, –SH, –COOH to improve arsenic binding), and magnetic or carbon supports (for improved dispersion and recovery) provides a rational design path toward next-generation materials. By uniting these design principles with scalable synthesis and durability testing under realistic water chemistries, UiO-66-derived adsorbents can evolve into robust, regenerable, and environmentally sustainable solutions for arsenic-contaminated water remediation.

## Figures and Tables

**Table 2:** Some list of different MOFs used for arsenic adsorption from water.

Adsorbents	Pollutants	Surface Area (BET) (m^2^ g^−1^)	$q_{max}$ * (mg g^−1^)	References
Fe-BTC	As(V)	-	12.3	Zhu et al. (2012) [105]
ZIF-8	As(V)	1388	76.5	Li et al. (2014a) [106]
ZIF-8	As(III)/As(V)	1064	49/60	Jian et al. (2015) [109]
MOF-74	As(V)	-	99	Tarboush et al. (2018) [107]
MIL-100(Fe)	As(III)/As(V)	720	35.2/19.2	Wang et al. (2018) [108]
MIL-53(Fe)	As(V)	14	21.3	Vu et al. (2015) [110]
MIL-101(Fe)	As(V)	1370	110	Cai et al. (2016) [111]
MIL-53(Al)	As(V)	920	105.6	Li et al. (2014b) [112]
MOF-808	As(V)	-	24.83	Li et al. (2015) [113]
UiO-66	As(V)	570	303.4 (147.7~303.4)	Wang et al. (2015) [23]
UiO-66	As(III)/As(V)	1318.23	110.47/132.18	Pandi et al. (2020) [114]
UiO-66	As(III)/As(V)	485.9	205/71.13	He et al. (2019) [115]

* Based on Langmuir Model.

**Table 3:** Some list of different UiO-66 adapted ligands used for arsenic adsorption from water.

Adsorbents	Pollutants	Surface Area (BET) (m^2^ g^−1^)	$q_{max}$ *** (mg g^−1^)	References
UiO-66-NH_2_	As(III)/As(V)	113.4	200/68.21	He et al. (2019) [115]
UiO-66-NH_2_	As(V)	468	76.9	Chang et al. (2019) [118]
* HP-UiO-66-NH_2_	As(V)	974.43	84.03 ~248.75	Xu et al. (2020) [119]
UiO-66-NH_2_	As(III)/As(V)	300	104.8/131.6	Somjit et al. (2022a) [120]
UiO-66-(SH) _2_	As(III)/As(V)	1150	40.0/10.0	Audu et al. (2016) [121]
UiO-66-(SH) _2_	Hg(II)	499	236.4	Leus et al. (2017) [122]
** am-UiO-66-NO_2_	As(V)	531	85	Wang et al. (2022) [123]
UiO-66-NO_2_	As(V)	660	68	Wang et al. (2022) [123]
UiO-66-EDTA	11 metals	383.5	Hg(II) 161.2 Pb(II) 153.8	Wu et al. (2019) [124]

* HP: Hierarchically porous, ** am: amorphous and *** Based on Langmuir Model.

**Table 4:** Regeneration and Structural Retention of UiO-66 Derivatives After Arsenic Adsorption Cycles.

Adsorbent	Pollutant(s)	BET * Surface Area (m^2^ g^−1^)	$q_{max}$ ** (mg/g)	Regeneration (Cycles/Retention) %	PXRD After Cycling	N_2_ Sorption After Cycling (Retained)	Reference
UiO-66–NH_2_–HCl	As(V)	468–855	76.9–161.3	5/90%	Peaks unchanged; minor intensity loss	92% BET	Chang et al. (2019) [118]
UiO-66–SH–Al^3+^	As(III)/As(V)	30.2	90.9/98.8	4/80%	Slight broadening; Partial oxidation	80% BET	Shao et al. (2019) [125]
UiO-66–TFA/AA	As(V)	1041–1690	89.3–365.4	6/95%	Stable lattice reflections	94% BET	Assaad et al. (2020) [126]
Lattice-Defected UiO-66 (t-ZrO_2_)	As(III)/As(V)	133.7	352.1/147.5	4/88%	Minor shift; defect-related broadening	87% BET	Qu et al. (2022) [128]
DU (Defected UiO-66)	As(III)	946–1238	204	5/91%	Well-preserved peaks	90% BET	Somjit et al. (2022b) [129]
UiO-66–DABA	Hg(II)	753.9–1329.2	713	6/96%	No peak shift; high crystallinity	95% BET	Zhao et al. (2023) [131]

Abbreviations: * BET = Brunauer–Emmett–Teller surface area; TFA = Trifluoroacetic acid; AA = Acetic acid; DU = Defected UiO-66; DABA = 3,5-Diaminobenzoic acid; PXRD = Powder X-ray Diffraction. ** Based on Langmuir Model.

**Table 5:** Some list of different UiO-66 adapted Modulator (defect) used for arsenic adsorption from water.

Adsorbents	Pollutants	Surface Area (BET) (m^2^ g^−1^)	$q_{max}$ **** (mg g^−1^)	References
UiO-66-NH_2_-HCl	As(V)	468–855	76.9–161.3	Chang et al. (2019) [118]
UiO-66-SH-A *	As(III)/As(V)	30.21	90.9/98.8	Shao et al. (2019) [125]
UiO-66-TFA **, AA ***	As(V)	1041–1690	89.3–365.4	Assaad et al. (2020) [126]
Lattice defected UiO-66 (t-ZrO_2_)	As(III)/As(V)	133.7	352.1/147.5	Qu et al. (2022) [128]
DU (defected UiO-66)	As(III)	946–1238	204	Somjit et al., (2022b) [129]
UiO-66-DABA (DABA: 3,5-diaminobenzoic acid)	Hg(II)	753.9– 1329.2	713	Zhao et al. (2023) [131]

* A: heating treatment at 900 °C, ** TFA: trifluoroacetic acid, *** AA: acetic acid and **** Based on Langmuir Model.

## Data Availability

No new data were created or analyzed in this study.

## Supplementary Materials

The following supporting information can be downloaded at https://www.mdpi.com/article/10.3390/nano15211621/s1: UiO-66 Membranes Applications. References [162,163,164,165,166] are cited in the supplementary materials.

## References

[1] Bissen M., Frimmel F.H. (2003). Arsenic—A review. Part I: Occurence, Toxicity, Speciation, Mobility. Acta Hydrochim. Hydrobiol..

[2] Payne K., Abdel-Fattah T.M. (2005). Adsorption of arsenate and arsenite by iron-treated activated carbon and zeolites: Effects of pH, temperature, and ionic strength. J. Environ. Sci. Health.

[3] Sarkar A., Paul B. (2016). The global menace of arsenic and its conventional remediation—A critical review. Chemosphere.

[4] Siddique T., Dutta N.K., Roy Choudhury N. (2020). Nanofiltration for Arsenic Removal: Challenges, Recent Developments, and Perspectives. Nanomaterials.

[5] Kanel S.R., Grenèche J.-M., Choi H. (2006). Arsenic(V) Removal from Groundwater Using Nano Scale Zero-Valent Iron as a Colloidal Reactive Barrier Material. Environ. Sci. Technol..

[6] Nicomel R.N., Leus K., Folens K., Van Der Voort P., Du Laing G. (2016). Technologies for Arsenic Removal from Water: Current Status and Future Perspectives. Int. J. Environ. Res. Public Health.

[7] Ferguson J.F., Gavis J. (1972). Review of Arsenic Cycle in Natural Waters. Water Res..

[8] Sorlini S., Gialdini F. (2010). Conventional oxidation treatments for the removal of arsenic with chlorine dioxide, hypochlorite, potassium permanganate and monochloramine. Water Res..

[9] Meng X., Bang S., Korfiatis G.P. (2000). Effects of silicate, sulfate, and carbonate on arsenic removal by ferric chloride. Water Res..

[10] Sato Y., Kang M., Kamei T., Magara Y. (2002). Performance of nanofiltration for arsenic removal. Water Res..

[11] Kim J., Benjamin M.M. (2004). Modeling a novel ion exchange process for arsenic and nitrate removal. Water Res..

[12] Tokunaga S. (1999). Removal of arsenic(III) and arsenic(V) ions from aqueous solutions with lanthanum(III) salt and comparison with aluminum(III), calcium(II), and iron(III) salts. Water Environ. Res..

[13] Mohan D., Pittman C.U. (2007). Arsenic removal from water/wastewater using adsorbents—A critical review. J. Hazard. Mater..

[14] Wang S., Peng Y. (2010). Natural Zeolites as Effective Adsorbents in Water and Wastewater Treatment. Chem. Eng. J..

[15] Yuan S., Feng L., Wang K., Pang J., Bosch M., Lollar C., Sun Y., Qin J., Yang X., Zhang P. (2018). Stable metal–organic frameworks: Design, synthesis, and applications. Adv. Mater..

[16] Yaghi O.M., Kalmutzki M.J., Diercks C.S. (2019). Introduction to Reticular Chemistry: Metal-Organic Frameworks and Covalent Organic Frameworks.

[17] Wang C., Luan J., Wu C. (2019). Metal-organic frameworks for aquatic arsenic removal. Water Res..

[18] Liu M.-Y., Zhang L., Li Y.-H., Wang C.-C., Wang P., Zhao C., Fu H. (2024). Defective NH_2_-UiO-66 for effective Pb(II) removal: Facile fabrication strategy, performances and mechanisms. Prog. Nat. Sci. Mater. Int..

[19] Chen W., Lin S.-Z., Song Z., Huang G.-B., Zhang M. (2025). Construction of S-scheme UiO-66-NH_2_/Zn_0.4_Cd_0.6_S hybrid architectures with strong interfacial interactions triggering efficient photocatalytic H_2_O_2_ production, nitrogen fixation and water splitting. J. Mater. Sci. Technol..

[20] Liang K., Guo W., Li L., Cai H., Zhang H., Li J., Xu F., Yan J., Lv D., Xi H. (2024). Defect-induced synthesis of nanoscale hierarchically porous metal–organic frameworks with tunable porosity for enhanced volatile organic compound adsorption. Nano Mater. Sci..

[21] Wang Y., Meng X. (2025). Single-Atom Cu Anchored on a UiO-66 Surface-Enhanced Raman Scattering Sensor for Trace and Rapid Detection of Volatile Organic Compounds. Research.

[22] Xu X., Xu Y., Zhang J., Zhong Y., Li Z., Qiu H., Wu H.B., Wang J., Wang X., Gu C. (2023). Quasi-Solid Electrolyte Interphase Boosting Charge and Mass Transfer for Dendrite-Free Zinc Battery. Nano-Micro Lett..

[23] Wang C., Liu X., Chen J.P., Li K. (2015). Superior removal of arsenic from water with zirconium metal-organic framework UiO-66. Sci. Rep..

[24] Cullen W.R. (2008). Is Arsenic an Aphrodisiac? The Sociochemistry of an Element.

[25] Smedley P.L., Kinniburgh D.G. (2002). A review of the source, behaviour and distribution of arsenic in natural waters. Appl. Geochem..

[26] Bardach A.E., Ciapponi A., Soto N., Chaparro M.R., Calderon M., Briatore A., Cadoppi N., Tassara R., Litter M.I. (2015). Epidemiology of chronic disease related to arsenic in Argentina: A systematic review. Sci. Total Environ..

[27] Chen C.J., Chuang Y.C., You S.L., Lin T.M., Wu H.Y. (1986). A retrospective study on malignant neoplasms of bladder, lung and liver in blackfoot disease endemic area in Taiwan. J. Cancer.

[28] Berg M., Tran H.C., Nguyen T.C., Pham H.V., Schertenleib R., Giger W. (2001). Arsenic contamination of groundwater and drinking water in Vietnam: A human health threat. Environ. Sci. Technol..

[29] Nickson R., McArthur J., Burgess W., Ahmed K.M., Ravenscroft P., Rahman M. (1998). Arsenic poisoning of Bangladesh groundwater. Nature.

[30] Fendorf S., Michael H.A., van Geen A. (2010). Spatial and temporal variations of groundwater arsenic in South and Southeast Asia. Science.

[31] Hughes M.F. (2002). Arsenic toxicity and potential mechanisms of action. Toxicol. Lett..

[32] EFSA (European Food Safety Authority) (2009). Scientific opinion on arsenic in food. EFSA J..

[33] Chakraborti D., Das B., Rahman M.M., Chowdhury U.K., Biswas B., Goswami A.B., Nayak B., Pal A., Sengupta M.K., Ahamed S. (2011). Status of groundwater arsenic contamination in the state of West Bengal, India: A 20-year study repor. Mol. Nutr. Food Res..

[34] WHO (World Health Organization) Arsenic in Drinking-Water: Background Document for Development of WHO Guidelines for Drinking-Water Quality. https://iris.who.int/server/api/core/bitstreams/ac5cc702-7983-4b6a-8094-869f4fe95593/content.

[35] Agency for Toxic Substances and Disease Registry (ATSDR), Addendum to the Toxicological Profile for Arsenic, Agency for Toxic Substances and Disease Registry Division of Toxicology and Human Health Sciences Atlanta. https://www.atsdr.cdc.gov/toxprofiles/Arsenic_addendum.pdf.

[36] USEPA (U.S. Environmental Protection Agency) National Primary Drinking Water Regulations. https://www.epa.gov/ground-water-and-drinking-water/national-primary-drinking-water-regulations.

[37] Sorg T.J., Logsdon G.S. (1978). Treatment Technology to Meet Interim Primary Drinking-Water Regulations for Inorganics: Part. 2. J. Am. Water Work. Assoc..

[38] Ghurye G., Clifford D. (2004). As(III) oxidation using chemical and solid-phase oxidants. J. Am. Water Work. Assoc..

[39] Ding W., Zheng H., Sun Y., Zhao Z., Zheng X., Wu Y., Xiao W. (2021). Activation of MnFe2O4 by sulfite for fast and efficient removal of arsenic (III) at circumneutral pH: In-volvement of Mn(III). J Hazard. Mater..

[40] Davis C.C., Edwards M. (2014). Coagulation With Hydrolyzing Metal Salts: Mechanisms and water Quality Impacts. Crit. Rev. Environ. Sci. Technol..

[41] Lakshmanan D., Clifford D., Samanta G. (2008). Arsenic removal by coagulation with aluminum, iron, titanium, and zirconium. J. Am. Water Work. Assoc..

[42] Bandaru S.R.S., van Genuchten C.M., Kumar A., Glade S., Hermandez D., Nahata M., Gadgil A. (2020). Rapid and Efficient Arsenic Removal by Iron Electrocoagulation Enabled with in Situ Generation of Hydrogen Peroxide. Environ. Sci. Technol..

[43] Nidheesh P.V., Anantha Singh A.T.S. (2017). Arsenic removal by electrocoagulation process: Recent trends and removal mechanism. Chemosphere.

[44] Kumar P.R., Chaudhari S., Khilar K.C., Mahajan S. (2004). Removal of arsenic from water by electrocoagulation. Chemosphere.

[45] Algieri C., Pugliese V., Coppola G., Curcio S., Calabro V., Chakraborty S. (2022). Arsenic removal from groundwater by membrane technology: Advantages, disadvantages, and effect on human health. Groundw. Sustain. Dev..

[46] Berg M., Luzi S., Trang P.T.K., Viet P.H., Giger W., Stüben D. (2006). Arsenic removal from groundwater by household sand filters: Comparative field study, model calculations, and health benefits. Environ. Sci. Technol..

[47] Voegelin A., Kaegi R., Berg M., Nitzsche K., Kappler A., Lan V.M., Trang P.T.K., Göttlicher J., Steininger R. (2014). Solid-phase characterisation of an effective household sand filter for As, Fe and Mn removal from groundwater in Vietnam. Environ. Chem..

[48] Chowdhury S.R., Yanful E.K. (2011). Arsenic removal from aqueous solutions by adsorption on magnetite nanoparticles. Water Environ. J..

[49] Peng X., Xi B., Zhao Y., Shi Q., Meng X., Mao X., Jiang Y., Ma Z., Tan W., Liu H. (2017). Effect of Arsenic on the Formation and Adsorption Property of Ferric Hydroxide Precipitates in ZVI Treatment. Environ. Sci. Technol..

[50] Zhu N.Y., Yan T.M., Qiao J., Cao H.L. (2016). Adsorption of arsenic, phosphorus and chromium by bismuth impregnated biochar: Adsorption mechanism and depleted adsorbent utilization. Chemosphere.

[51] Sun X.F., Hu C., Hu X.X., Qu J.H., Yang M. (2013). Characterization and adsorption performance of Zr-doped akaganéite for efficient arsenic removal. J. Chem. Technol. Biotechnol..

[52] Farha O.K., Eryazici I., Jeong N.C., Hauser B.G., Wilmer C.E., Sarjeant A.A., Snurr R.Q., Nguyen S.T., Yazaydin A.Ö., Hupp J.T. (2012). Metal–Organic Framework Materials with Ultrahigh Surface Areas: Is the Sky the Limit?. J. Am. Chem. Soc..

[53] Su C., Puls R. (2001). Arsenate and arsenite removal by zero-valent iron: Kinetics, redox transformation and implications for in situ groundwater remediation. Environ. Sci. Technol..

[54] Zhang N., Eric M., Zhang C., Zhang J., Feng K., Li Y., Wang S. (2021). ZVI impregnation altered arsenic sorption by ordered mesoporous carbon in presence of Cr(VI): A mechanistic investigation. J. Hazard. Mater..

[55] Lorenzen L., van Deventer J.S.J., Land W.M. (1995). Factors affecting the mechanism of the adsorption of arsenic species on activated carbon. Miner. Eng..

[56] Liu Z., Zhang F.S., Sasai R. (2010). Arsenate removal from water using Fe_3_O_4_-loaded activated carbon prepared from waste biomass. Chem. Eng. J..

[57] Bang S., Patel M., Lippincott L., Meng X. (2005). Removal of arsenic from ground water by granular titanium dioxide adsorbent. Chemosphere.

[58] Maiti A., Basu J.K., De S. (2010). Development of a Treated Laterite for Arsenic Adsorption: Effects of Treatment Parameters. Ind. Eng. Chem. Res..

[59] Habuda-Stanic M., Kalajdžić B., Kuleš M., Velić N. (2008). Arsenite and arsenate sorption by hydrous ferric oxide/polymeric material. Desalination.

[60] Jian N., Maiti A. (2022). Fe-Mn-Al metal oxides/oxyhydroxides as As(III) oxidant under visible light and adsorption of total arsenic in the groundwater environment. Sep. Purif. Technol..

[61] Nakamoto K., Kobayashi T. (2019). Arsenate and arsenite adsorbents composed of nano-sized cerium oxide deposited on activated alumina. Sep. Sci. Technol..

[62] Yean S., Cong L., Yavuz C.T., Mayo J.T., Yu W.W., Kan A.T., Colvin V.L., Tomson M.B. (2005). Effect of magnetite particle size on adsorption and desorption of arsenite and arsenate. J. Mater. Res..

[63] Liu C.H., Chuang Y.H., Chen T.Y., Tian Y., Wang M.-K., Zhamg W. (2015). Mechanism of arsenic adsorption on magnetite nanoparticles from water: Thermodynamic and spectroscopic studies. Environ. Sci. Technol..

[64] Gupta A.R., Joshi V.C., Yadav A., Sharma S. (2022). Synchronous Removal of Arsenic and Fluoride from Aqueous Solution: A Facile Approach to Fabricate Novel Functional Metallopolymer Microspheres. ACS Omega.

[65] Cui H., Li Q., Gao S., Shang J.K. (2012). Strong adsorption of arsenic species by amorphous zirconium oxide nanoparticles. J. Ind. Eng. Chem..

[66] Setyono D., Valiyaveettil S. (2014). Chemically modified sawdust as renewable adsorbent for arsenic removal from water. ACS Sustain. Chem. Eng..

[67] Han Y.S., Kim S.H., Jang J.Y., Ji S. (2022). Arsenic removal characteristics of natural Mn-Fe binary coating on waste filter sand from a water treatment facility. Environ. Sci. Pollut. Res..

[68] Hao L., Zheng T., Jiang J., Hu Q., Li X., Wang P. (2015). Removal of As (III) from water using modified jute fibres as a hybrid adsorbent. RSC Adv..

[69] Feng L., Cao M., Ma X., Zhu Y., Hu C. (2012). Superparamagnetic high-surface-area Fe_3_O_4_ nanoparticles as adsorbents for arsenic removal. J. Hazard. Mater..

[70] Czaja A.U., Trukhan N., Müller U. (2009). Industrial applications of metal-organic frameworks. Chem. Soc. Rev..

[71] Jiao L., Seow J.Y.R., Skinner W.S., Wang Z.U., Jiang H.L. (2019). Metal-organic frameworks: Structures and functional applications. Mater. Today.

[72] Cmarik G.E., Kim M.K., Cohen S.M., Walton K.S. (2012). Tuning the adsorption properties of UiO-66 via ligand functionalization. Langmuir.

[73] Wang C., Liu X., Keser Demir N., Chen J.P., Li K. (2016). Applications of water stable metal–organic frameworks. Chem. Soc. Rev..

[74] Kinoshita Y., Matsubara I., Higuchi T., Saito Y. (1959). The crystal structure of bis(adiponitrilo) copper(I) nitrate. Bull. Chem. Soc. Jpn..

[75] Hoskins B.F., Robson R. (1989). Infinite polymeric frameworks consisting of three dimensionally linked rod-like segments. J. Am. Chem. Soc..

[76] Yaghi O.M., Li G., Li H. (1995). Selective Binding and Removal of Guests in a Microporous Metal-Organic Framework. Nature.

[77] Yaghi O.M., Sun Z., Richardson D.A., Groy T.L. (1994). Directed Transformation of Molecules to Solids: Synthesis of a Microporous Sulfide from Molecular Germanium Sulfide Cages. J. Am. Chem. Soc..

[78] Yaghi O.M., Li H. (1995). Hydrothermal Synthesis of a Metal-Organic Framework Containing Large Rectangular. Channels J. Am. Chem. Soc..

[79] Furukawa H., Cordova K., Michael O., Yaghi O.M. (2013). The Chemistry and Applications of Metal-Organic Frameworks. Science.

[80] Bennett T.D., Tan J.C., Yue Y., Baxter E., Ducati C., Terrill N.J., Yeung H.H., Zhou Z., Chen W., Henke S. (2015). Hybrid glasses from strong and fragile metal-organic framework liquids. Nat. Commun..

[81] Férey G., Serre C., Mellot-Draznieks C., Millange F., Surblé S. (2004). A Hybrid Solid with Giant Pores Prepared by a Combination of Targeted Chemistry, Simulation, and Powder Diffraction. Angew. Chem..

[82] Cavka J.H., Jakobsen S., Olsbye U., Guillou N., Lamberti C., Bordiga S., Lillerud K.P. (2008). A New Zirconium Inorganic Building Brick Forming Metal Organic Frameworks with Exceptional Stability. J. Am. Chem. Soc..

[83] Feng M., Zhang P., Zhou H.-C., Sharma V.K. (2018). Water-stable metal-organic frameworks for aqueous removal of heavy metals and radionuclides: A review. Chemosphere.

[84] Zhang X., Wang B., Alsalme A., Xiang S., Zhang Z., Chen B. (2020). Design and applications of water-stable metal-organic frameworks: Status and challenges. Coord. Chem. Rev..

[85] Winarta J., Shan B., Mcintyre S.M., Ye L., Wang C., Liu J., Mu B. (2020). A Decade of UiO-66 Research: A Historic Review of Dynamic Structure, Synthesis Mechanisms, and Characterization Techniques of an Archetypal Metal–Organic Framework. Cryst. Growth Des..

[86] Ghosh P., Colón Y.J., Snurr R.Q. (2014). Water Adsorption in UiO-66: The Importance of Defects. Chem. Commun..

[87] Chen Z., Hanna S.L., Redfern L.R., Alezi D., Islamoglu T., Farha O.K. (2019). Reticular chemistry in the rational synthesis of functional zirconium cluster-based MOFs. Coord. Chem. Rev..

[88] Zou D., Liu D. (2019). Understanding the modifications and applications of highly stable porous frameworks via UiO-66. Mater. Today Chem..

[89] Kuwahara Y., Kango H., Yamashita H. (2017). Catalytic Transfer Hydrogenation of Biomass-Derived Levulinic Acid and Its Esters to γ-Valerolactone over Sulfonic Acid-Functionalized UiO-66. ACS Sustain. Chem. Eng..

[90] Homaee M., Hamadi H., Nobakht V., Javaherian M., Salahshournia B. (2019). Ultrasound-assisted synthesis of UiO-66-NHSO_3_H via post-synthetic modification as a heterogeneous Brønsted acid catalyst. Polyhedron.

[91] Moghadaskhou F., Eivazzadeh-Keihan R., Sadat Z., Tadjarodi A., Maleki A. (2023). Fabrication and characterization of a novel catalyst based on modified zirconium metal-organic-framework for synthesis of polyhydroquinolines. Sci. Rep..

[92] Edubilli S., Gumma S. (2019). A systematic evaluation of UiO-66 metal organic framework for CO_2_/N_2_ separation. Sep. Purif. Technol..

[93] Kazemi A., Moghadaskhou F., Pordsari M.A., Manteghi F., Tadjarodi A., Ghaemi A. (2023). Enhanced CO_2_ capture potential of UiO-66-NH_2_ synthesized by sonochemical method: Experimental findings and performance evaluation. Sci. Rep..

[94] Chavan S., Vitillo J.G., Gianolio D. (2012). H_2_ storage in isostructural UiO-67 and UiO-66 MOFs. Phys. Chem. Chem. Phys..

[95] Zhao Q., Yuan W., Liang J., Li J. (2013). Synthesis and hydrogen storage studies of metal–organic framework UiO-66. Int. J. Hydrogen Energy.

[96] Ren J., Langmi H.W., North B.C., Mathe M., Bessarabov D. (2014). Modulated synthesis of zirconium-metal organic framework (Zr-MOF) for hydrogen storage applications. Int. J. Hydrogen Energy.

[97] Ma Y., Lu W., Han X., Chen Y., da Silva I., Lee D., Sheveleva A.M., Wang Z., Li J., Li W. (2022). Direct Observation of Ammonia Storage in UiO-66 Incorporating Cu(II) Binding Sites. J. Am. Chem. Soc..

[98] Lázaro I.A., Wells C.J.R., Forgan R.S. (2020). Multivariate Modulation of the Zr MOF UiO-66 for Defect-Controlled Combination Anticancer Drug Delivery. Angew. Chem..

[99] Strauss I., Chakarova K., Mundstock A., Mihaylov M., Hadjiivanov K., Guschanski N., Caro J. (2020). UiO-66 and UiO-66-NH_2_ based sensors: Dielectric and FTIR investigations on the effect of CO_2_ adsorption. Microporous Mesoporous Mater..

[100] Zhai Z., Zhang X., Wang J., Li H., Sun Y., Hao X., Qin Y., Niu B., Li C. (2022). Washable and flexible gas sensor based on UiO-66-NH_2_ nanofibers membrane for highly detecting SO_2_. Chem. Eng. J..

[101] Min X., Wu X., Shao P., Ren Z., Ding L., Luo X. (2019). Ultrahigh capacity of lanthanum-doped UiO-66 for phosphate capture: Unusual doping of lanthanum by the reduction of coordination number. Chem. Eng. J..

[102] Athari M., Fattahi M., Khosravi-Nikou M., Hajhariri A. (2022). Adsorption of different anionic and cationic dyes by hybrid nanocomposites of carbon nanotube and graphene materials over UiO-66. Sci. Rep..

[103] Ahmadijokani F., Molavi H., Rezakazemi M., Tajahmadi S., Bahi A., Ko F., Aminabhavi T.M., Li J.-R., Arjmand M. (2022). UiO-66 metal–organic frameworks in water treatment: A critical review. Prog. Mater. Sci..

[104] Cheng S., Xie P., Yu Z., Gu R., Su Y. (2022). Enhanced Adsorption Performance of UiO-66 via Modification with Functional Groups and Integration into Hydrogels. Environ. Res..

[105] Zhu B.-J., Yu X.-Y., Jia Y., Peng F.-M., Sun B., Zhang M.-Y., Luo T., Liu J.-H., Huang X.-J. (2012). Iron and 1,3,5-Benzenetricarboxylic Metal–Organic Coordination Polymers Prepared by Solvothermal Method and Their Application in Efficient As(V) Removal from Aqueous Solutions. J. Phys. Chem. C.

[106] Li J., Wu Y., Li Z., Zhang B., Zhu M., Hu X., Zhang Y., Li F. (2014). Zeolitic Imidazolate Framework-8 with High Efficiency in Trace Arsenate Adsorption and Removal from Water. J. Phys. Chem. C.

[107] Tarboush B.J., Chouman A., Jonderian A., Ahmad M., Hmadeh M., Al-Ghoul M. (2018). Metal–Organic Framework-74 for Ultratrace Arsenic Removal from Water: Experimental and Density Functional Theory Studies. ACS Appl. Nano Mater..

[108] Wang D., Gilliland S.E., Yi X., Logan K., Heitger D.R., Lucas H.R., Wang W.-N. (2018). Iron Mesh-Based Metal Organic Framework Filter for Efficient Arsenic Removal. Environ. Sci. Technol..

[109] Jian M., Liu B., Zhang G., Liu R., Zhang X. (2015). Adsorptive removal of arsenic from aqueous solution by zeolitic imidazolate framework-8 (ZIF-8) nanoparticles. Colloids Surf. A Physicochem. Eng. Asp..

[110] Vu T.A., Le G.H., Dao C.D., Dang L.Q., Nguyen Q.K., Dang P.T., Tran H.T.K., Duong Q.T., Nguyen T.V., Lee G.D. (2015). Arsenic removal from aqueous solution by adsorption using novel MiL-53(Fe) as highly efficient adsorbent. RSC Adv..

[111] Cai J., Wang X., Zhou Y., Jiang L., Wang C. (2016). Selective adsorption of arsenate and the reversible structure transformation of the mesoporous metal-organic framework MIL-100(Fe). Phys. Chem. Chem. Phys..

[112] Li J., Wu Y., Li Z., Zhu M., Li F. (2014). Characteristics of arsenate removal from water by metal-organic frameworks (MOFs). Water Sci. Technol..

[113] Li Z.-Q., Yang J.-C., Sui K.-W., Yin N. (2015). Facile synthesis of metal-organic framework MOF-808 for arsenic removal. Mater. Lett..

[114] Pandi K., Lee D.W., Choi J. (2020). Facile synthesis of zirconium-organic frameworks@biomass-derived porous graphitic nanocomposites: Arsenic adsorption performance and mechanism. J. Mol. Liq..

[115] He X., Deng F., Shen T., Yang L., Chen D., Luo J., Luo X., Min X., Wang F. (2019). Exceptional adsorption of arsenic by zirconium metal-organic frameworks: Engineering exploration and mechanism insight. J. Colloid. Interface Sci..

[116] Islamoglu T., Otake K.-i., Li P., Buru C.T., Peters A.W., Akpinar I., Garibay S.J., Farha O. (2018). Revisiting the structural homogeneity of NU-1000, a Zr-based metal–organic framework. Cryst. Eng. Comm..

[117] Furukawa H., Gándara F., Zhang Y.-B., Jiang J., Queen W.L., Hudson M.R., Yaghi O.M. (2014). Water Adsorption in Porous Metal-Organic Frameworks and Related Materials. J. Am. Chem. Soc..

[118] Chang Z.-W., Lee Y.-J., Lee D.-J. (2019). Adsorption of hydrogen arsenate and dihydrogen arsenate ions from neutral water by UiO-66-NH_2_. J. Environ. Manag..

[119] Xu R., Ji Q., Zhao P., Jian M., Xiang C., Hu C., Zhang G., Tang C., Liu R., Zhang X. (2020). Hierarchically porous UiO-66 with tunable mesopores and oxygen vacancies for enhanced arsenic removal. J. Mater. Chem. A.

[120] Somjit V., Thinsoongnoen P., Sriphumrat K., Pimu S., Arayachukiat S., Kongpatpanich K. (2022). Metal-Organic Framework Aerogel for Full pH Range Operation and Trace Adsorption of Arsenic in Water. ACS Appl. Mater. Interfaces.

[121] Audu C.O., Nguyen H.G.T., Chang C.Y., Katz M.J., Mao L., Farha O.K., Hupp J.T., Nguyen S.T. (2016). The dual capture of As(V) and As(III) by UiO-66 and analogues. Chem. Sci..

[122] Leus K., Perez J.P.H., Folens K., Meledina M., Van Tendeloo G., Du Laing G., Van Der Voort P. (2017). UiO-66-(SH)_2_ as stable, selective and regenerable adsorbent for the removal of mercury from water under environmentally-relevant conditions. Faraday Discuss..

[123] Wang X., Lyu Q., Tong T., Sun K., Lin L.-C., Tang C.Y., Yang F., Guiver M.D., Quan X., Dong Y. (2022). Robust ultrathin nanoporous MOF membrane with intra-crystalline defects for fast water transport. Nat. Commun..

[124] Wu J., Zhou J., Zhang S., Alsaedi A., Hayat T., Li J., Song Y. (2019). Efficient removal of metal contaminants by EDTA modified MOF from aqueous solutions. J. Colloid. Interface Sci..

[125] Shao P., Ding P.L., Luo J., You D., Zhang Q., Luo X. (2019). Lattice-Defect-Enhanced Adsorption of Arsenic on Zirconia Nanospheres: A Combined Experimental and Theoretical Study. ACS Appl. Mater. Interfaces.

[126] Assaad N., Sabeh G., Hmadeh M. (2020). Defect Control in Zr-Based Metal−Organic Framework Nanoparticles for Arsenic Removal from Water. ACS Appl. Nano Mater..

[127] Kandiah M., Nilsen M.H., Usseglio S., Jakobsen S., Olsbye U., Tilset M., Larabi C., Quadrelli E.A., Bonio F., Lillerud K.P. (2010). Synthesis and Stability of Tagged UiO-66 Zr-MOFs. Chem. Mater..

[128] Qu G., Jia P., Zhang T., Li Z., Chen C., Zhao Y. (2022). UiO-66(Zr)-derived t-zirconia with abundant lattice defect for remarkably enhanced arsenic removal. Chemosphere.

[129] Somjit V., Thinsoongnoen P., Pila T., Boekfa B., Wannapaiboon S., Kongpatpanich K. (2022). Hydroxylation of UiO-66 Metal–Organic Frameworks for High Arsenic(III) Removal Efficiency. Inorg. Chem..

[130] Shen L., Liang R., Luo M., Jing F., Wu L. (2015). Electronic effects of ligand substitution on metaleorganic framework photocatalysts: The case study of UiO-66. Phys. Chem. Chem. Phys..

[131] Zhao C., Yang G., Zhang S., He X., Zhong Y., Gao X. (2023). Enhanced Breathing Effect of Nanoporous UIO-66-DABA Metal–Organic Frameworks with Coordination Defects for High Selectivity and Rapid Adsorption of Hg(II). ACS Appl. Nano Mater..

[132] Davydiuk T., Chen X., Huang L., Shuai Q., Le X.C. (2020). Removal of inorganic arsenic from water using metal organic frameworks. J. Environ. Sci..

[133] Shan B., James J.B., Armstrong M.R., Close E.C., Letham P.A., Nikkhah K., Lin Y.S., Mu B. (2018). Influences of Deprotonation and Modulation on Nucleation and Growth of UiO-66: Intergrowth and Orienta-tion. J. Phys. Chem. C.

[134] Zhao Y., Zhang Q., Li Y., Zhang R., Lu G. (2017). Large-Scale Synthesis of Monodisperse UiO-66 Crystals with Tunable Sizes and Missing Linker Defects via Acid/Base Co-Modulation. ACS Appl. Mater. Interfaces.

[135] Vermoortele F., Bueken B., Le Bars G., Vande Voorde B., Vandichel M., Houthoofd K., Vimont A., Daturi M., Waroquier M., VanSpeybroeck V. (2013). Synthesis Modulation as a Tool To Increase the Catalytic Activity of Metal-Organic Frameworks: The Unique Case of UiO-66(Zr). J. Am. Chem. Soc..

[136] Sun J., Zhang X., Zhang A., Liao C. (2019). Preparation of Fe–Co based MOF-74 and its effective adsorption of arsenic from aqueous solution. J. Environ. Sci..

[137] Feng C., Aldrich C., Eksteen J.J., Arrigan D.W.M. (2017). Removal of arsenic from alkaline process waters of gold cyanidation by use of γ-Fe_2_O_3_@ZrO_2_ nanosorbents. Hydrometallurgy.

[138] Yang J.C., Yin X.B. (2017). CoFe_2_O_4_@MIL-100(Fe) hybrid magnetic nanoparticles exhibit fast and selective adsorption of arsenic with high adsorption capacity. Sci. Rep..

[139] Pervez N., Chen C., Li Z., Naddeo V., Zhao Y. (2022). Tuning the structure of cerium-based metal-organic frameworks for efficient removal of arsenic species: The role of organic ligands. Chemosphere.

[140] Liu T.Y., Zhang Z.C., Wang Z.H., Wang Z.L., Bush R. (2019). Highly efficient and rapid removal of arsenic(iii) from aqueous solutions by nanoscale zero-valent iron supported on a zirconium 1,4-dicarboxybenzene metal–organic framework (UiO-66 MOF). RSC Adv..

[141] Picchi D.F., Biglione C., Horcajada P. (2024). Nanocomposites Based on Magnetic Nanoparticles and Metal-Organic Frame works for Therapy, Diagnosis, and Theragnostics. ACS Nanosci. Au.

[142] Wu M.-X., Wang Y., Zhou G., Liu X. (2020). Core–Shell MOFs@MOFs: Diverse Designability and Enhanced Selectivity. ACS Appl. Mater. Interfaces.

[143] Chen R., Tao C., Zhang Z., Chen X., Liu Z., Wang J. (2019). Layer-by-Layer Fabrication of Core-Shell Fe3O4@UiO-66-NH2 with High Catalytic Reactivity toward the Hydrolysis of Chemical Warfare Agent Simulants. ACS Appl. Mater. Interfaces.

[144] Qi P., Luo R., Pichler T., Zeng J., Wang Y., Fan Y., Sui K. (2019). Development of a magnetic core-shell Fe_3_O_4_@TA@UiO-66 microsphere for removal of arsenic(III) and antimony(III) from aqueous solution. J. Hazard. Mater..

[145] Huo J.-B., Xu L., Chen X., Zhang Y., Yang J.-C.E., Yuan B., Fu M.-L. (2019). Direct epitaxial synthesis of magnetic Fe_3_O_4_@UiO-66 composite for efficient removal of arsenate from water. Microporous Mesoporous Mater..

[146] Ahmadipouya S., Haris M.H., Ahmadijokani F., Jarahiyan A., Molavi H., Moghaddam F.M., Rezakazemi M., Arjmand M. (2021). Magnetic Fe_3_O_4_@UiO-66 nanocomposite for rapid adsorption of organic dyes from aqueous solution. J. Mol. Liq..

[147] Ahmadijokani F., Tajahmadi S., Haris M.H., Bahi A., Rezakazemi M., Molavi H., Ko F., Arjmand M. (2021). Fe_3_O_4_@PAA@UiO-66-NH2 magnetic nanocomposite for selective adsorption of Quercetin. Chemosphere.

[148] Singh S., Naik T.S.S.K., U B., Khan N.A., Wani A.B., Behera S.K., Nath B., Bhati S., Sigh J., Ramamurthy P.C. (2022). A systematic study of arsenic adsorption and removal from aqueous environments using novel graphene oxide functionalized UiO-66-NDC nanocomposites. Sci. Rep..

[149] Boix G., Troyano J., Garzon-Tovar L., Camur C., Bermejo N., Yazdi A., Piella J., Bastus N.G., Puntes V.F., Imaz I. (2020). MOF-Beads Containing Inorganic Nanoparticles for the Simultaneous Removal of Multiple Heavy Metals from Water. ACS Appl. Mater. Interfaces.

[150] Jiang Z.-R., Li Y., Zhang D., Zhou Y.-X., Xu G., Wang C., Lan Y., Guo J. (2021). Decorating S-doped Cu-La bimetallic oxides with UIO-66 to increase the As(III) adsorption capacity via synchronous oxidation and adsorption. J. Hazard. Mater..

[151] Guo Q., Li Y., Zheng L.-W., Xu Y., Shen Y.-W., Zhang K.-G., Yuan C.-G. (2023). Facile fabrication of Fe/Zr binary MOFs for arsenic removal in water: High capacity, fast kinetics and good reusability. J. Environ. Sci..

[152] Li Z., Ma S., Sang L., Qu G., Zhang T., Xu B., Jin W., Zhao Y. (2023). Enhanced Arsenite Removal from Water Using Zirconium-Ferrocene MOFs Coupled with Peroxymono sulfate: Oxidation and Multi-sites Adsorption Mechanism. Chemosphere.

[153] Zhu G., Lin J., Yuan Q., Wang X., Zhao Z., Hursthouse A.S., Wang Z., Li Q. (2021). A biochar supported magnetic metal organic framework for the removal of trivalent antimony. Chemosphere.

[154] Obeidli A., Salah H.B., Murisi M., Sabouni R. (2022). Recent advancements in MOFs synthesis and their green applications. Int. J. Hydrogen Energy.

[155] Hao F., Yan X.-P. (2022). Nano-sized zeolite-like metal-organic frameworks induced hematological effects on red blood cell. J. Hazard. Mater..

[156] Wiśniewska P., Haponiuk J., Saeb M.R., Rabiee N., Bencherif S.A. (2023). Mitigating metal-organic framework (MOF) toxicity for biomedical applications. Chem. Eng. J..

[157] Kumar P., Anand B., Tsang Y.F., Kim K.-H., Khullar S., Wang B. (2019). Regeneration, degradation, and toxicity effect of MOFs: Opportunities and challenges. Environ. Res..

[158] Ashour R.M., Abdel-Magied A.F., Wu Q., Olsson R.T., Forsberg K. (2020). Green Synthesis of Metal-Organic Framework Bacterial Cellulose Nanocomposites for Separation Applications. Polymers.

[159] An H.T., Zhang X., Dong C., Lu M.-Y., Li R., Xie Y., Xie L.-H., Li J.-R. (2023). Seed-aided green synthesis of metal-organic frameworks in water. Green. Chem. Eng..

[160] Venturi D.M., Campana F., Marmottini F., Costantino F., Vaccaro L. (2020). Extensive Screening of Green Solvents for Safe and Sustainable UiO-66 Synthesis. ACS Sustain. Chem. Eng..

[161] Biehler E., Pagola S., Stam D., Merkelbach J., Jandl C., Abdel-Fattah T.M. (2025). A Comparison of Microcrystal Electron Diffraction and X-ray Powder Diffraction for the Structural Analysis of Metal–Organic Frameworks. Appl. Crystallogr..

[162] Fonseca J., Gong T. (2022). Fabrication of metal-organic framework architectures with macroscopic size: A review. Co-ord. Chem. Rev..

[163] Liu X., Demir N.K., Wu Z., Li K. (2015). Highly Water-Stable Zirconium Metal–Organic Framework UiO-66 Membranes Supported on Alumina Hollow Fibers for Desalination. J. Am. Chem. Soc..

[164] Wang Y., Wang S., Fang J., Ding L.-X., Wang H. (2017). A nano-silica modified polyimide nanofiber separator with enhanced thermal and wetting properties for high safety lithium-ion batteries. J. Membr. Sci..

[165] Eltaweil A.S., Mamdouh I.M., El-Monaem E.M.A., El-Subruiti G.M. (2021). Highly Efficient Removal for Methylene Blue and Cu^2+^ onto UiO-66 Metal–Organic Framework/Carboxylated Graphene Oxide-Incorporated Sodium Alginate Beads. ACS Omega.

[166] Singh H., Goyal A., Bhardwaj S.K., Khatri M., Bhardwaj N. (2022). Highly robust UiO-66@PVDF metal–organic framework beads for tartrazine removal from aqueous solutions. Mater. Sci. Eng. B.

