# Supplementary material for: Advancing Arsenic Water Treatment Using UiO-66 and Its Functionalized Metal–Organic Framework Analogs
Source: Nanomaterials (Basel). 2025 Oct 24;15(21):1621. doi: 10.3390/nano15211621 (PMC12608536; doi:10.3390/nano15211621)
Supplement: Supplementary file 1 [file nanomaterials-15-01621-s001.zip › nanomaterials-3917967-supplementary.pdf]

Supplementary Information

Advancing Arsenic Water Treatment Using UiO-66 and Its Functionalized Metal–Organic Framework Analogs

Sangwoo Ji 1 and Tarek M. Abdel-Fattah 2*

1 Geo-Environmental Research Division, Korea Institute of Geoscience and Mineral Resources (KIGAM), 124 Gwahak-ro, Yuseong-gu, Daejeon 34132, Republic of Korea; swji@kigam.re.kr (S.J.)

2 Applied Research Center at Thomas Jefferson National Accelerator Facility and Department of Biology, Chemistry and Environmental Science, Christopher Newport University, Newport News, VA 23606, USA

*Correspondence: fattah@cnu.edu (T.A.F.)

UiO-66 Membranes Applications

MOFs are typically produced as polydisperse microcrystalline powders. The industrial applicability of MOF powders is limited by their mechanical, chemical, and attrition resistance, mass transfer limitations, or poor handling properties [1]. The introduction of magnetic properties offers one solution to this problem, with researchers developing macroscopic architectures for industrial-level implementation.

One of the other solutions is fabricated UiO-66 Membranes ([2-4] produced UiO-66 polycrystalline membranes on alumina hollow fibers using an in situ solvothermal synthesis method. Single-gas permeation and ion rejection tests confirmed membrane integrity and functionality. The membrane exhibited excellent rejection of multivalent ions (e.g., 86.3% for Ca^{2+}, 98.0% for Mg^{2+}, and 99.3% for Al^{3+}) based on size exclusion, with moderate permeance (0.14 L m^{-2} h^{-1} bar^{-1}) and good permeability (0.28 L L m^{-2} h^{-1} bar^{-1}μm). In He et al.'s research (2017) [3], three water-stable zirconium metal-organic framework (MOF) UiO-66 nanoparticles with diameters of 30, 100, and 500 nm were synthesized and incorporated into the selective layer to form thin-film nanocomposite (TFN) membranes. Compared to thin-film composite (TFC) membranes, TFN membranes exhibited higher pure water permeability (PWP) and rejections for pollutants due to their smaller pore size and higher hydrophilicity. The TFN membrane comprising 30 nm UiO-66 demonstrated the best performance among the three TFN membranes. Additionally, the TFN membrane exhibited robust long-term stability. Wang et al. (2022) [4] fabricated an ultrathin missing-linker UiO-66 (ML-UiO-66) membrane for water desalination. They employed a molecular-level intra-crystalline defect strategy combined with a selective layer-thinning protocol to enable fast water permeation. Besides almost complete salt rejection, high and stable water flux was achieved even under long-term pervaporation operations in harsh environments, effectively addressing challenging stability issues.

UiO-66 beads also could be one option [5-6]. Eltaweil et al. (2021) [5] synthesized a MOF-based composite beads adsorbent by incorporating UiO-66 MOF and carboxylated graphene oxide (GOCOOH) into sodium alginate (UiO-66/GOCOOH@SA). They utilized UiO-66/GOCOOH@SA for the removal of methylene blue dye and Cu^{2+} ions. Singh et al. (2023) [6] developed UiO-66@PVDF composite beads using the phase inversion method, with polyvinylidene fluoride (PVDF) containing 50% MOF by weight. This composite achieved a remarkable 97% removal of tartrazine dye from aqueous solutions. Somjit et al. (2022a) [7] synthesized UN aerogel composites (MOF-Alginate) incorporating UiO-66-NH_2 and alginate. The UN aerogel exhibited excellent acid-base tolerance across the entire pH spectrum (pH from 1 to 14) and effectively removed trace amounts of arsenic from real samples, achieving the Maximum Contaminant Level Goal (MCLG) of arsenic species in drinking water, set at 0 ppb. Furthermore, the UN aerogel demonstrated markedly higher breakthrough adsorption capacity and longer lifespan compared to other arsenic decontamination adsorbents.

References

1. Fonseca, J.; Gong, T. Fabrication of metal-organic framework architectures with macroscopic size: A review. *Coord. Chem. Rev.* **2022**, *462*, https://doi.org/10.1016/j.ccr.2022.214520.
2. Liu, X.; Demir, N.K.; Wu, Z.; Li, K. Highly Water-Stable Zirconium Metal–Organic Framework UiO-66 Membranes Supported on Alumina Hollow Fibers for Desalination. *J. Am. Chem. Soc.* **2015**, *137*, 6999–7002, https://doi.org/10.1021/jacs.5b02276.
3. Wang, Y.; Wang, S.; Fang, J.; Ding, L.-X.; Wang, H. A nano-silica modified polyimide nanofiber separator with enhanced thermal and wetting properties for high safety lithium-ion batteries. *J. Membr. Sci.* **2017**, *537*, 248–254, https://doi.org/10.1016/j.memsci.2017.05.023.
4. Wang, X.; Lyu, Q.; Tong, T.; Sun, K.; Lin, L.-C.; Tang, C.Y.; Yang, F.; Guiver, M.D.; Quan, X.; Dong, Y. Robust ultrathin nanoporous MOF membrane with intra-crystalline defects for fast water transport. *Nat. Commun.* **2022**, *13*, 1–11, https://doi.org/10.1038/s41467-021-27873-6.
5. Eltaweil, A.S.; Mamdouh, I.M.; El-Monaem, E.M.A.; El-Subruiti, G.M. Highly Efficient Removal for Methylene Blue and Cu^{2+} onto UiO-66 Metal–Organic Framework/Carboxylated Graphene Oxide-Incorporated Sodium Alginate Beads. *ACS Omega* **2021**, *6*, 23528–23541, https://doi.org/10.1021/acsomega.1c03479.
6. Singh, H.; Goyal, A.; Bhardwaj, S.K.; Khatri, M.; Bhardwaj, N. Highly robust UiO-66@PVDF metal–organic framework beads for tartrazine removal from aqueous solutions. *Mater. Sci. Eng. B* **2022**, *288*, https://doi.org/10.1016/j.mseb.2022.116165.
7. Somjit, V.; Thinsoongnoen, P.; Sriphumrat, K.; Pimu, S.; Arayachukiat, S.; Kongpatpanich, K. Metal–Organic Framework Aerogel for Full pH Range Operation and Trace Adsorption of Arsenic in Water. *ACS Appl. Mater. Interfaces* **2022**, *14*, 40005–40013, https://doi.org/10.1021/acsami.2c10664.
